# Mitigating Cellular Dysfunction Through Contaminant Reduction in Synthetic circRNA for High‐Efficiency mRNA‐Based Cell Reprogramming

**DOI:** 10.1002/advs.202416629

**Published:** 2025-03-05

**Authors:** Ziwei Zhang, Weiyu Li, Xiangyu Ren, Dengwang Luo, Xiushuang Yuan, Li Yu, Daming Wang, Yuhong Cao

**Affiliations:** ^1^ CAS Key Laboratory for Biological Effects of Nanomaterials and Nanosafety National Center for Nanoscience and Technology Chinese Academy of Sciences Haidian District Beijing 100190 China; ^2^ Sino‐Danish College University of Chinese Academy of Sciences Huairou District Beijing 100190 China; ^3^ College of Nanoscience and Technology University of Chinese Academy of Sciences Huairou District Beijing 100049 China; ^4^ Frontier Science Center for Synthetic Biology and Key Laboratory of Systems Bioengineering (Ministry of Education) School of Chemical Engineering and Technology Tianjin University Jinnan District Tianjin 300072 China; ^5^ Division of Life Sciences and Medicine University of Science and Technology of China Hefei Anhui 230001 China; ^6^ BiosynRNA Biotechnology Company Haidian District Beijing 100192 China

**Keywords:** cell reprogramming, circular RNA, iPSC, mRNA therapy, purification strategy

## Abstract

Synthetic circular RNA (circRNA) holds great promise for biomedical research and therapeutic applications, but impurities introduced during synthesis trigger innate immune responses and significantly compromise its efficacy. In this study, key immunogenic byproducts, including double‐stranded RNA, 5′ triphosphates from uncircularized RNA, and hydrolyzed RNA fragments, are identified as impairing circRNA functionality via RNA‐sensing pathways. To address this, a multi‐step purification process is developed that combines enzymatic treatments and cellulose‐based filtration to effectively remove these contaminants. This approach significantly reduces immune activation and increases manufacturing yields of circRNA by over 10‐fold. The purified circRNA demonstrates exceptional performance in induced pluripotent stem cells (iPSCs) reprogramming, achieving over 300% reprogramming efficiency with just three OSKMLN circRNA transfection treatments. Additionally, the purified circRNA achieves high levels and persistent expression of chimeric antigen receptor (CAR) in T cells with high cytotoxicity against tumor cells, making it a promising candidate for mRNA‐based CAR‐T cell therapy. These findings establish the purification strategy as a scalable and reliable platform for producing therapeutic‐grade RNA, with broad applications in mRNA‐based cell reprogramming for regenerative medicine and cancer immunotherapy.

## Graphical abstract

This study addresses the challenges of synthetic circular RNAimpurities that trigger immune responses and hinder efficacy. Key immunogenic byproducts are identified and develop a multi‐step purification process that produces ultra‐pure circRNA and enhances yields by over 10‐fold. The purified circRNA demonstrates remarkable reprogramming efficiency in iPSCs and high cytotoxicity in T cells, highlighting its potential for mRNA‐based reprogramming.

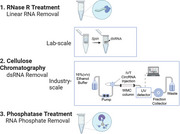

## 1 Introduction

Circular RNA (circRNA) is a type of RNA that forms a covalently closed loop, playing crucial roles in regulating gene expression and cellular processes.^[^ ^1^, ^2^, ^3^, ^4^, ^5^ ^]^ Recently, it has emerged as a promising therapeutic agent in fields such as cancer immunotherapy and vaccine development due to its unique structural properties.^[^ ^6^, ^7^, ^8^, ^9^, ^10^, ^11^ ^]^ Compared to linear mRNA, circRNA is relatively resistant to exonuclease degradation, resulting in enhanced stability. This stability allows for prolonged expression in target cells, making circRNA an ideal candidate for therapeutic applications.^[^ ^12^ ^]^

Previous studies have shown that mRNA‐based reprogramming offers several advantages, including low genotoxicity, high efficiency, and versatile delivery methods.^[^ ^13^, ^14^ ^]^ This process involves introducing synthetic mRNAs into target cells to translate proteins that induce pluripotency or direct differentiation. However, the rapid decay of synthetic mRNA often limits the effectiveness of this approach. In contrast, circRNA offers a compelling alternative as a result of its prolonged expression and stability, which can enhance the efficiency of cell reprogramming by maintaining protein levels over extended periods. Despite these benefits, the immunogenicity of circRNA remains controversial, as it may trigger immune responses that compromise cellular functions. Additionally, challenges in the synthesis and purification of circRNA can result in impurities that adversely affect its functionality and safety.^[^ ^12^, ^15^, ^16^, ^17^ ^]^ Quality control methods, particularly ion‐paired reversed‐phase high‐performance liquid chromatography (IP‐RP HPLC), have been established to identify and characterize impurities in synthetic circular RNA, which can significantly impact the biological activities and overall efficacy.^[^ ^18^ ^]^ Addressing these issues is essential for unlocking the full potential of circRNA in the application of cell reprogramming.

The production of synthetic circRNA generates various immunogenic byproducts during in vitro transcription (IVT) and RNA circularization.^[^ ^15^ ^]^ These impurities can activate multiple RNA‐sensing pathways, including Toll‐like receptors (TLRs) and RNA‐dependent protein kinase R (PKR), leading to immune responses that compromise RNA stability and expression.^[^ ^19^, ^20^, ^21^, ^22^, ^23^, ^24^, ^25^, ^26^, ^27^ ^]^ Current purification methods, such as RNase R digestion and size‐exclusion liquid chromatography (SEC), are ineffective at removing structured or linear byproducts, resulting in immune activation and reduced protein expression.^[^ ^15^, ^28^ ^]^ While denaturing urea polyacrylamide gel electrophoresis can separate circRNA, it is unsuitable for large‐scale preparation. Therefore, optimized purification strategies are essential for producing high‐quality circRNA for therapeutic applications, making the development of scalable and efficient methods critical to realizing its full therapeutic potential.

In this work, we systematically analyzed the immunogenicity of RNA byproducts generated during circRNA synthesis and identified four key contaminants: RNA precursors, nicked RNA, double‐stranded RNA (dsRNA), and RNA with 5′ triphosphate groups. Based on our findings, we developed a stepwise purification strategy that combines enzymatic digestion with cellulose filtration. This method effectively removes immune‐activating impurities, substantially reduces immune responses, and enhances circRNA expression, achieving superior manufacturing yields compared to conventional approaches. Most importantly, the purified circRNA demonstrated exceptional functionality and minimal immunogenicity in advanced therapeutic applications. In induced pluripotent stem cells (iPSCs) reprogramming and T cell engineering, our purified circRNA exhibited outstanding performance, highlighting its potential as a robust platform for regenerative medicine and cancer immunotherapy. These results establish our purification method as a promising approach for producing therapeutic grade circRNA suitable for medical applications.

## 2 Results

### 2.1 Byproducts in Synthetic circRNA Significantly Trigger Innate Immune Responses

In the synthesis of circRNA, transcription from a DNA template is followed by the circularization of linear precursors through either direct ligation with T4 DNA/RNA ligase or self‐splicing via group I/II autocatalytic introns.^[^ ^6^, ^29^ ^]^ The immunogenicity of circRNA can stem from the formation of dsRNA byproducts, uncircularized RNA (precursor), or nicked RNA molecules, all of which are potent triggers of cellular immune mechanisms (Figure S1, Supporting Information).

**Figure 1:**
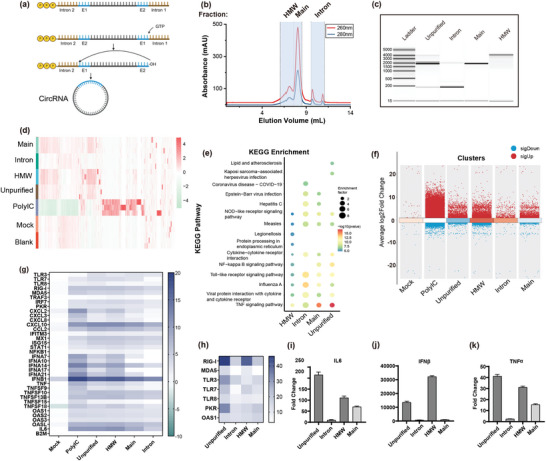
Innate immune activation by byproducts of IVT‐synthesized circular RNA. a) Schematic representation of RNA circularization with group I autocatalytic introns (Created with BioRender.com). b) Size exclusion chromatography separation of unpurified circRNA. c) Capillary electrophoresis of different fractions, as described in Figure 1b. d) Gene expression profiles of PMA‐differentiated THP‐1 cells transfected with different fractions (Data presented n = 3). e) Multiple KEGG enrichment analyses of PMA differentiated THP‐1 transfected with different fractions, with untreated cells serving as the control group. f) Multiple volcano plots illustrating transcriptomic differences between THP‐1 cells transfected with different fractions. Untreated cells served as the control group. Each cluster represents the comparison of a specific fraction‐transfected condition against the control. g) Genes associated with innate immune response were identified as differentially expressed candidate mRNAs upon unpurified, intron, early and HMW transfection relative to untransfected cells (n = 3). B2 m mRNA was used as a housekeeping control. h) Induction of RIG‐I, TLR3/7/8, MDA5, PKR, and OAS1 transcripts 6 h post‐transfection of PMA‐differentiated THP‐1 with unpurified, intron, main, and HMW fractions relative to untransfected cells (n = 3). B2 m mRNA was used as a housekeeping control. i–k) Induction of IFNβ, TNFα, and IL6 transcripts 6 h post‐transfection of PMA‐differentiated THP‐1 with unpurified, intron, main, and HMW relative to untransfected cells, B2 m mRNA was used as a housekeeping control. Data presented as means ± standard deviations (SDs), n = 3.

To identify the immunogenic RNA byproducts in synthesis circRNA, we employed a circRNA encoding enhanced green fluorescent protein (circ‐eGFP) according to ^(1)^ via group I intron‐based method. (**Figure** **1**a). Capillary electrophoresis and IP‐RP HPLC analysis of the unpurified circRNA preparation revealed four distinct peaks: the low molecular weight peaks for excised introns of the circularization process, a main circRNA peak that contained the desired circRNA, a precursor peak, and a high molecular weight (HMW) peak (Figures S2a and S2c, Supporting Information). To explore the immunogenicity of different byproducts, we employed SEC to fractionate the unpurified circ‐eGFP into distinct components, including HMW, main, and intron fractions (Figure 1b). As shown in Figure 1c, the intron fraction contained introns and a small proportion of the main peak, whereas the main fraction contained circRNA, nicked RNA, and linear precursors. The HMW fraction primarily consisted of HMW byproducts such as dsRNA and other RNA aggregates.

We then transfected unpurified circRNA, main fraction, HMW, or intron fraction, into THP‐1 cells to examine their immunogenicity in cells (Methods). Transcriptome analysis revealed that cells treated with unpurified circRNA, as well as those treated with the main, intron, and HMW fractions, all exhibited significant changes in gene expression patterns (Figure 1d–f).

Kyoto Encyclopedia of Genes and Genomes (KEGG) pathway enrichment analysis further identified significant differences in immune response pathways between blank and various RNA fractions, with enriched pathways including TNF, TLR, NF‐κB signaling, and cytokine–cytokine receptor interactions. The unpurified cricRNA fraction showed the highest enrichment factors and significant *p*‐values, indicating strong pathway associations. The main fraction exhibited similar enrichment, likely due to linear precursors and nicked RNA. Despite lower transfection amounts, intron and HMW fractions also showed notable enrichment. We also identified genes upregulated by unpurified circRNA (Figure 1g), revealing significant activation of RNA sensors (TLR3/7/8, PKR, OAS, MDA5, RIG‐I) in unpurified, main, and HMW fractions, leading to cytokine and interferon production. The potent induction of IFNβ by the HMW fraction can likely be attributed to the high levels of dsRNA.^[^ ^30^, ^31^, ^32^ ^]^ RT‐qPCR results (Figure 1h–k) further confirmed these findings, showing that unpurified circ‐eGFP and HMW fractions had the highest induction of RNA sensors and cytokines, likely due to immunogenic RNA species such as dsRNA.

These results reveal the heterogeneous nature of unpurified circRNA, suggesting that trace synthesis‐derived impurities can activate innate immune pathways and impair circRNA functionality, underscoring the need for effective purification strategies to ensure therapeutic safety and efficacy.

### 2.2 Removal of Immunogenic Contaminants to Enhance circRNA Performance

**Figure 2:**
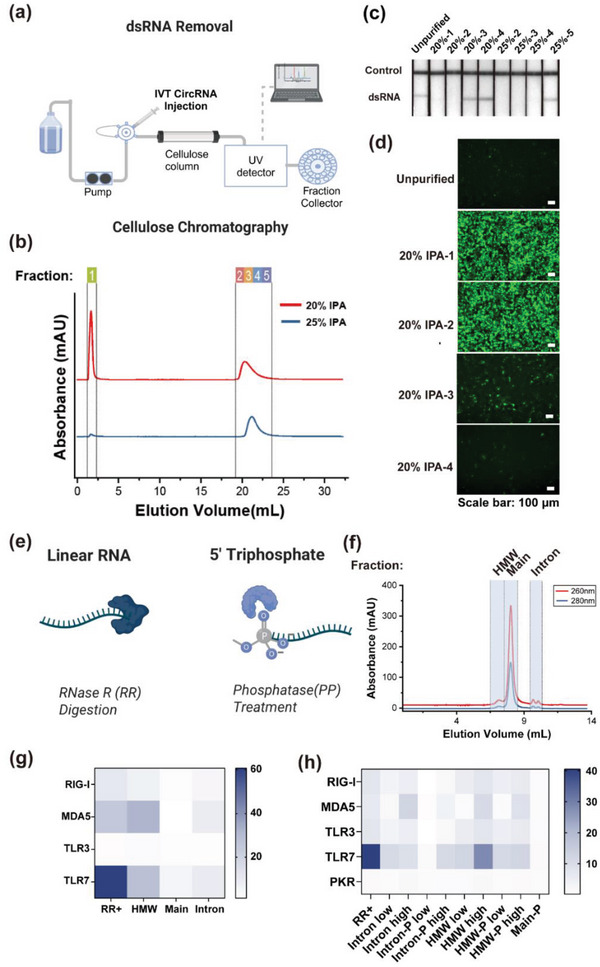
Removal of immunogenic contaminants enhances circRNA translation efficiency and reduces immune response. a) Purification of IVT circRNA using MCC chromatography to eliminate dsRNA. (Created with BioRender.com). b) MCC chromatogram of unpurified circ‐eGFP utilizing 20% (red) and 25% (blue) isopropanol (IPA)‐HEPES equilibrium conditions. c) LFSA assessment of circRNA fractions as outlined in Figure 2b. d) Representative cellular fluorescence image 24 h post‐transfection of HeLa cells with different fractions (scale bar: 100 µm). e) Enzymatic treatment for immunogenic contaminant removal. (Created with BioRender.com). f) SEC separation of RR+ circ‐eGFP. g) Induction of RIGI, TLR3, TLR7, and MDA5 transcripts 6 h after transfection of A549 cells with the 40 ng of the intron, HMW, or 200 ng of a main fraction of SEC separated RR+circ‐eGFP (*n* = 3). B2 m mRNA was used as a housekeeping control. h) Induction of RIG‐I, TLR3, TLR7, and MDA5 transcripts 6 h after transfection of A549 cells with the 40 or 200 ng of intron and HMW, before and after phosphatase treatment (intron‐P, HMW‐P), or 200 ng of main fraction after phosphatase treatment (main‐P) (*n* = 3). B2 m mRNA was used as a housekeeping control.

Our findings indicate that trace high molecular weight (HMW) impurities, including dsRNA, can negatively impact circRNA functionality. To address this, we employed microcrystalline cellulose (MCC) chromatography to evaluate its efficacy in removing HMW impurities that adversely affect circRNA performance and protein expression (**Figure** **2**a).^[^ ^32^ ^]^ Using an isopropanol (IPA)‐HEPES buffer system, we collected both the flow‐through and subsequent elution peaks (Figure 2b). Capillary electrophoresis revealed distinct RNA distributions: introns were primarily in the flow‐through and early elution peaks, while HMW species were mainly in the later elution peaks (Figure S3a–c and Table S1, Supporting Information). A lateral flow strip assay (LFSA) confirmed the absence of dsRNA in the early elution peaks, indicating successful removal (Figure 2c).^[^ ^33^ ^]^ Protein expression tests in HeLa cells showed that the initial elution peaks significantly enhanced protein expression and transfection efficiency, while later fractions exhibited diminished effects (Figure 2d; Figure S3d, Supporting Information). Notably, the flow‐through fraction with the highest intron content also increased protein expression, underscoring the negative impact of HMW impurities, which were inversely correlated with protein expression outcomes (R^2^ = 0.84, Figure S3e, Supporting Information).

To further enhance circRNA purity, we investigated enzymatic treatments to address immunogenic contaminants (Figure 2e). We subjected unpurified circ‐eGFP to RR treatment, which selectively degrades linear RNA while preserving circRNA. RR digestion achieved ≈89.8% circ‐eGFP purity (Figure S2b–d, Supporting Information). However, the resulting protein expression levels were lower than those of samples treated solely for dsRNA removal (Figure S4, Supporting Information). Testing RR‐treated circ‐eGFP in A549 cells revealed that dsRNA‐containing fractions elicited strong immune responses, while the RR‐treated main fraction, largely free of linear contaminants, showed reduced immunogenicity (Figure 2g,h).

To address the immunogenicity of intron and HMW fractions, we treated them with phosphatase to remove 5′ triphosphate groups, known triggers for immune sensors like RIG‐I. Transfection into A549 cells showed that while phosphatase treatment reduced the immunogenicity of intron‐derived impurities, it had little effect on the immune response from HMW fractions, primarily containing dsRNA (Figure S5, Supporting Information). The main fraction also exhibited low immunogenicity after phosphatase digestion, attributed to the removal of the remaining 5′ triphosphates.

### 2.3 Combined Strategy Removes Immunogenic Byproducts and Yields High‐Purity circRNA

**Figure 3:**
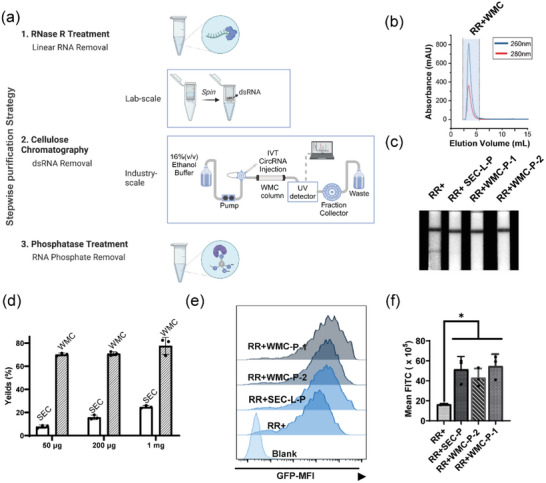
An optimized purification strategy yields high‐purity circRNA with enhanced efficiency. a) Illustration of our optimized purification strategy (Created with BioRender.com). b) WMC separation of 200 µg of RR+circ‐eGFP using FPLC. c) LFSA test of RR+, RR+SEC‐L‐P, RR+WMC‐P‐1 and RR+WMC‐P‐2. circ‐eGFP. d) Recovery rates following RR+SEC and RR+WMC (mean ± SD, n = 3). e,f) Protein expression levels of PBMCs 24 h post‐transfection with purified fractions (scale bar: 100 µm; One‐way ANOVA, ^*^ *p* < 0.05).

We have identified several immunogenic byproducts in IVT‐synthesized circRNA, including dsRNA in HMW fractions, 5′ triphosphate introns, precursors, and nicked RNA. Effective removal of these impurities is critical to producing high‐purity, low‐immunogenicity circRNA, which is essential for ensuring optimal performance and safety in advanced therapeutic applications. To confirm that the contaminants are the major source of immunogenicity, we refined our purification protocol by integrating MCC with enzymatic treatments (**Figure** **3**a). This approach aimed to achieve even higher purity levels of circRNA while mitigating immunogenic responses.

We purified RR+circ‐eGFP using MCC, which was subsequently treated with phosphatase (RR+CC‐P). Capillary electrophoresis and LFSA results confirmed the effective removal of dsRNA from RR+CC‐P circ‐eGFP, with only a minor fraction of introns (0.8%) and HMW components (2.1%) remaining (Figures S6a, S7a, and S8, Supporting Information). For comparison, a stepwise process involving RR digestion, SEC, and phosphatase treatment was used as a control. Both the early and late peaks from SEC (RR+SEC‐E‐P, RR+SEC‐L‐P) were collected (Figure S9, Supporting Information). Capillary electrophoresis and HPLC results confirmed the high purity of both RR+SEC‐E‐P and RR+SEC‐L‐P, although RR+SEC‐E‐P showed a slight increase in linear RNA. The RR+SEC‐L‐P fraction, obtained from the later SEC peak, was used as an ultra‐pure circRNA positive control (Figure S8, Supporting Information; Experimental Section ^[^ ^15^ ^]^).

We then evaluated purification efficacy by testing circRNA obtained through these different strategies. Protein expression in HeLa levels of RR+CC‐P circ‐eGFP were comparable to those of RR+SEC‐L‐P (ultra‐pure circRNA), demonstrating its effectiveness (Figure S7b–d, Supporting Information). In contrast, RR+SEC‐E‐P performed suboptimally, likely due to its enrichment in linear RNA contaminants.

Given these findings, we sought to further enhance the removal of immunogenic dsRNA while improving purification efficiency and scalability, a critical goal for large‐scale production. To achieve this, we introduced wood‐derived macroporous cellulose (WMC), a natural porous material, for the removal of dsRNA. The porous nature of WMC allows for efficient removal of dsRNA from circRNA of varying lengths while maintaining a high recovery rate of the target RNA on both laboratory‐ and industry‐scale.^[^ ^34^ ^]^

We evaluated the recovery efficiency of WMC chromatography using varying amounts of circ‐eGFP RNA (50, 200 µg, and 1 mg, Figure 3a,b). The 50 µg sample was purified using a micro‐separation column, while the 200 µg and 1 mg samples were processed using FPLC. Across all tested conditions, WMC completely removed dsRNA and achieved significantly higher recovery rates (>70%) compared to the conventional SEC‐based approach (Figure 3c,d), with particularly notable improvements at smaller input amounts (50 and 200 µg).

Following WMC or SEC purification, circ‐eGFP samples were treated with phosphatase, yielding final products designated as ultra‐pu6re: RR+WMC‐P‐1 (FPLC) and RR+WMC‐P‐2 (micro‐separation column), respectively. Purity assessments using capillary electrophoresis and HPLC confirmed that both WMC‐based methods produced high‐purity circRNA with negligible impurities (Figures S7 and S8, Supporting Information). Protein expression levels from WMC‐purified samples were comparable to those obtained using conventional protocols in HeLa cells and PBMCs (Figure 3e,f; Figure S10, Supporting Information). These results indicate that WMC is an effective method for purifying diverse circRNA species, offering clear advantages in recovery rate and scalability compared to SEC‐based methods.

### 2.4 Purified circRNA Displays Minimal Immunogenicity

**Figure 4:**
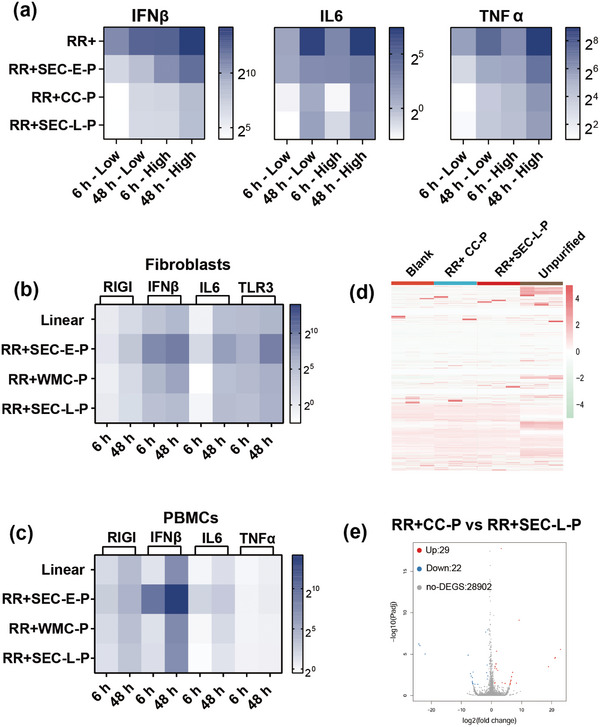
Purified circRNA exhibits minimal immunogenicity. a) Expression levels of inflammatory cytokines (IFNβ, TNFα, and IL6) in PMA‐differentiated THP‐1 cells at 6‐ and 48‐h post‐transfection with circ‐eGFP (200 ng or 1 µg) purified by different methods. Data normalized to B2 m and presented as mean ± SDs (*n* = 3). b) Expression levels of RIGI, IFNβ, IL6, and TLR3 in human primary fibroblasts at 6 and 48 h post‐transfection with circ‐eGFP purified by different methods. Data are normalized to B2m and presented as mean ± SD (*n* = 3). c) Expression levels of RIGI, IFNβ, IL6, and TNFα in PBMCs at 6 and 48 h post‐transfection with circ‐eGFP purified by different methods. Data are normalized to B2m and presented as mean ± SD (*n* = 3). d) Gene expression profiles of PMA‐differentiated THP‐1 cells transfected with indicated circ‐eGFP (*n* = 3). e) The volcano plot shows the difference in transcriptome between THP‐1 cells transfected with RR+SEC‐L‐P and RR+CC‐P circ‐eGFP.

Our studies demonstrate that circRNA purified using the RR+CC‐P method achieves protein expression levels comparable to those obtained through HPLC methods (e.g., RR+SEC‐L‐P). To explore the immunogenicity of our purified circRNA, we transfected THP‐1 cells with circ‐eGFP and a 5moU‐modified linear eGFP. Transcriptional analysis revealed that RR+CC‐P circ‐eGFP exhibited significantly reduced immunogenicity, with levels comparable to those of RR+SEC‐L‐P (**Figure** **4**a–d; Figure S11, Supporting Information). In contrast, RR+SEC‐E‐P showed relatively higher immunogenicity, likely due to linear contaminants, which cannot be completely separated by SEC.

RNA sequencing experiments comparing unpurified, ultra‐pure, and RR+CC‐P circRNA further confirmed the low immunogenicity of our purified circRNA (Figure 4d,e). Both the RR+CC‐P and ultra‐pure (RR+SEC‐L‐P) circRNA groups exhibited minimal differential gene expression compared to untreated controls, while unpurified circRNA induced significant transcriptomic changes, particularly in innate immune response pathways.

**Figure 5:**
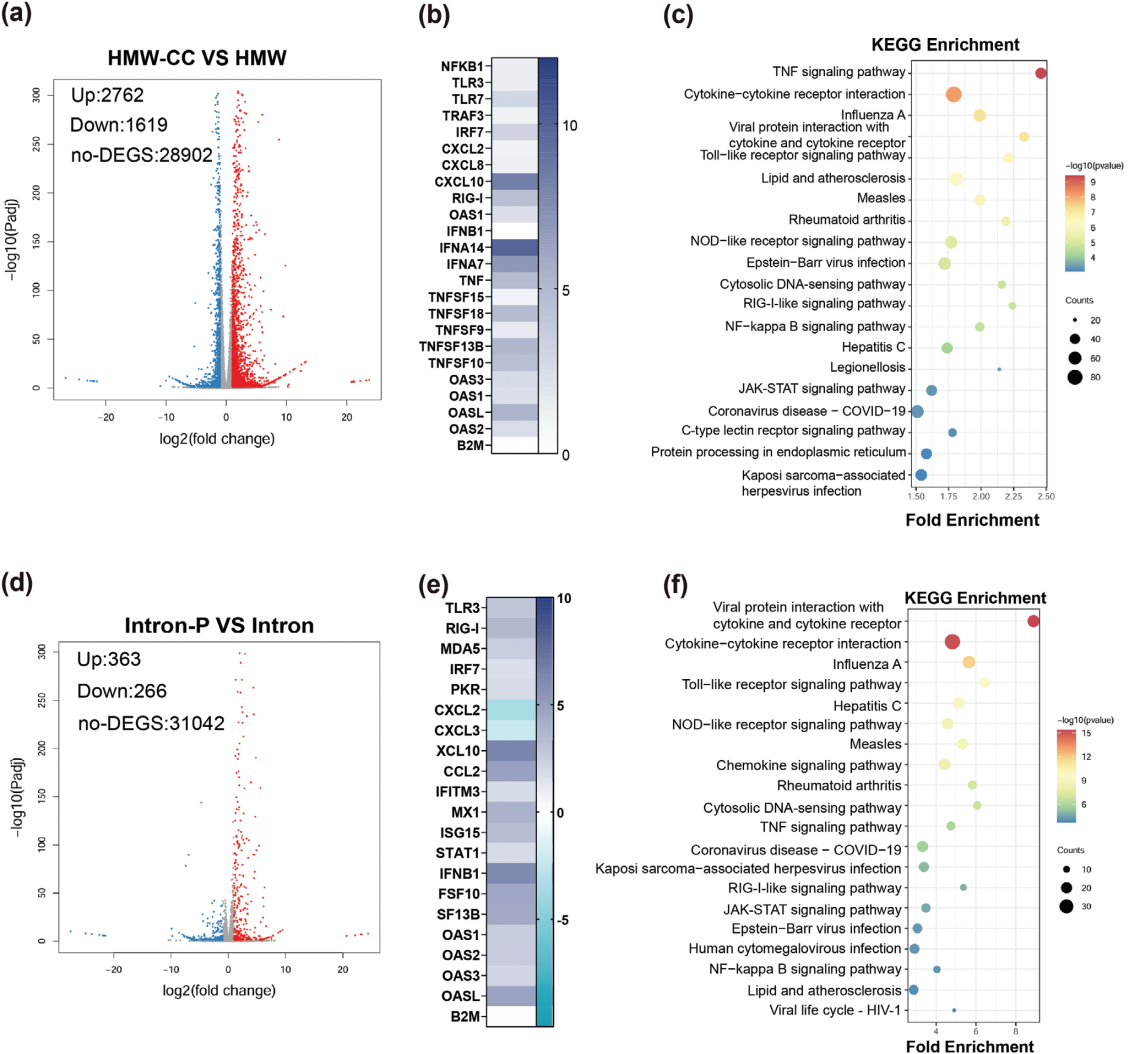
RNA Sequencing reveals residual HMW species and introns do not induce significant immunogenicity. a) The volcano plot shows the difference in transcriptome between THP‐1 cells transfected with HMW relative to the HMW after MCC chromatography (HMW‐CC) treated cells. b) Genes associated with innate immune response were identified as differentially expressed candidate mRNAs upon HMW transfection relative to HMW‐CC treated cells (*n* = 3). B2 m mRNA was used as a housekeeping control. c) KEGG enrichment analysis of PMA differentiated THP‐1 transfected with HMW‐CC, and HMW. d) The volcano plot shows the difference in transcriptome between THP‐1 cells transfected with intron‐P relative to the intron treated cells. e) Genes associated with innate immune response were identified as differentially expressed candidate mRNAs upon intron transfection relative to intron‐P treated cells (*n* = 3). B2 M mRNA was used as a housekeeping control. f) KEGG enrichment analysis of PMA differentiated THP‐1 transfected with intron and intron‐P.

Despite achieving high purity and low immunogenicity with RR+CC‐P circRNA, trace amounts of non‐dsRNA HMW impurities and introns persisted. To further evaluate the influence of the remaining contaminants, we conducted a differential expression analysis. The results showed that cells treated with HMW impurities before MCC resulted in 2762 upregulated and 1619 downregulated genes compared to treatment after MCC (**Figure** **5**a). Differentially expressed genes related to immune responses were identified (Figure 5b), with KEGG and GO analyses showing significant associations with TNF signaling, cytokine interactions, and TLR signaling (Figure 5c; Figure S12, Supporting Information). Thus, while dsRNA removal reduces immune responses, residual impurities, possibly the dimer form of circ‐eGFP, may still play a role.

For intron fractions, transcriptome analysis indicated 363 genes were upregulated and 266 downregulated before and after phosphatase treatments (Figure 5d,e). KEGG and GO analyses (Figure 5f; S13a, Supporting Information) showed these genes were mainly involved in the innate immune response. RT‐qPCR confirmed decreased mRNA levels of RNA molecular sensors and cytokines post‐phosphatase treatment (Figure S13b,c, Supporting Information). Overall, these findings highlight the effectiveness of our purification strategy in reducing immunogenic responses.

### 2.5 Purified circRNA Enabling High‐Efficiency iPSCs Reprogramming

Our findings demonstrate that even trace amounts of impurities can significantly impact protein expression and cellular function. Thus, achieving high‐purity circRNA is essential for optimal outcomes and preventing dysfunction related to circRNA impurities. To evaluate the efficacy of our highly purified circRNA, we conducted cellular reprogramming experiments using circRNA derived from the transcription factors OSKMLN (Oct4, Sox2, Klf4, cMyc, Nanog, and Lin28A).

**Figure 6:**
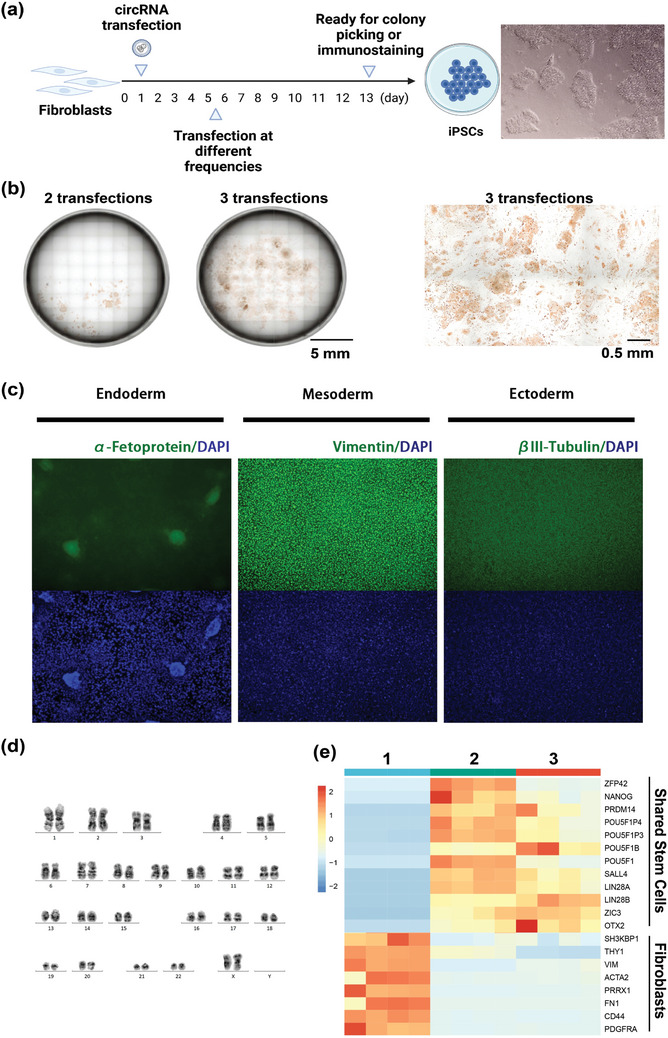
Functional validation of purified circRNA in cell reprogramming. a) A schematic diagram illustrating the induction of iPSCs using circRNA (Created with BioRender.com). b) Representative images of TRA‐1‐60‐stained reprogramming wells, showing the yield of TRA‐1‐60‐positive colonies on day 14 under the RR+WMC‐P circRNA‐based reprogramming regimen with varying transfection frequencies, initiated with a plating density of 1000 cells. c) Immunofluorescence analysis of the three germ layers differentiated from the indicated iPSC lines showing histology and marker expression specific to ectoderm (βIII‐tubulin (TUJ1)), mesoderm (vimentin), and endoderm (α‐Fetoprotein). d) Karyotyping of indicated iPSC lines. e) RNA sequencing of fibroblasts, ^(1)^ iPSCs induced by the RR+WMC‐P circRNA, ^(2)^ and H9 iPSC positive controls, ^(3)^ with data shown as the mean of four biological replicates.

The activation of inflammatory pathways is one of the key factors in causing reprogramming failure.^[^ ^35^, ^36^ ^]^ Previous studies have shown that mRNA‐based approaches achieve high‐efficiency reprogramming as a result of low immunogenicity and high transfection efficiency.^[^ ^13^ ^]^ Our experiments revealed that transfection of RR+WMC‐P circRNA at varying frequencies from day 1 to day 7 resulted in the efficient generation of iPSC colonies with as few as two transfections (**Figure** **6**a,b). Notably, the group receiving three transfections achieved over 3000 iPSC colonies, corresponding to a reprogramming efficiency exceeding 300%, a level typically only attainable with modified mRNA.^[^ ^13^ ^]^ We also observed that iPSC‐like colonies emerged earlier in groups transfected with ultra‐pure circRNA (day 12, Figure S14, Supporting Information).

To investigate transcriptional heterogeneity within the iPSC population, we performed RNA‐Seq analysis on four randomly selected lines of the circRNA‐induced iPSC colonies. The resulting transcriptomic profiles were compared with two control groups: the parental fibroblast population and the well‐characterized human embryonic stem cell line H9. As illustrated in Figure 6e, the iPSCs generated via circRNA reprogramming exhibit a global gene expression pattern consistent with that observed in human embryonic stem cells, particularly with respect to key pluripotency markers.^[^ ^13^, ^37^, ^38^ ^]^ Moreover, there was a downregulation of fibroblast identity genes, indicating successful repression of the somatic transcriptional program and acquisition of a pluripotent state.

In contrast, the suboptimal circRNA (RR+SEC‐E‐P) failed to induce the fibroblast‐to‐iPSC transition under identical conditions (1000 cells seeded, 2 or 3 transfections), resulting in no iPSC colonies. While increasing the number of transfections could yield some iPSCs, the efficiency remained significantly lower (data not shown), likely due to the high immunogenicity of the suboptimal circRNA (Figure 4b). Elevated immunogenicity can lead to altered cell fate decisions, ultimately resulting in reprogramming failure.

These results underscore the critical importance of utilizing high‐purity circRNA to create a conducive environment for successful reprogramming and to ensure the generation of high‐quality iPSCs. Furthermore, our purification strategy minimizes the risk of unwanted cellular stress responses, enhancing the overall stability and functionality of the reprogrammed cells. Ultimately, our findings highlight that circRNA purity is a key factor in optimizing reprogramming protocols for biomedical applications.

### 2.6 Purified circRNA Enabling Enhanced T cell Engineering

**Figure 7:**
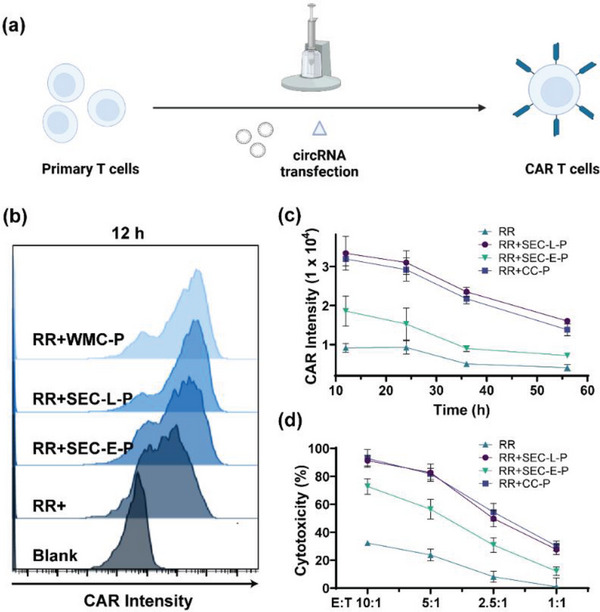
Functional validation of purified circRNA in T cell engineering. a) Schematic illustrating CAR‐T cell engineering via circRNA (Created with BioRender.com). b,c) CAR expression profiles in T cells transfected with circRNA purified by various methods, with data presented as the mean of three biological replicates. d) In vitro cytotoxicity of CAR‐T cell products generated from circRNA purified by various methods, following co‐culture with Raji‐luc cells, demonstrating a range of effects based on the effector‐to‐target (E:T) ratio, with data shown as the mean of three biological replicates.

We subsequently tested our purified circRNA in a challenging cellular application: T cell engineering. The use of high‐purity, low‐immunogenic gene vectors is crucial in this context, as it minimizes unwanted immune responses that can compromise T cell function and efficacy. We evaluated the efficacy of WMC‐purified anti‐human CD19 circ‐CAR in modifying T cells (**Figure** **7**a). Transcriptional analysis has revealed that WMC‐purified circ‐CAR elicited minimal innate immune responses (Figure 4c) while maintaining significant protein expression, comparable to ultra‐pure reference samples. Flow cytometric analysis further confirmed that T cells transfected with WMC‐purified circ‐CAR exhibited sustained CAR expression levels comparable to those of ultra‐pure circ‐CAR (RR+SEC‐L‐P), significantly surpassing the expression levels observed with RR+ and RR+SEC‐E‐P (Figure 7b,c).

Following this, we conducted in vitro cytotoxicity assays using Raji‐luc as the target cells, demonstrating that CAR‐T cells derived from the purified group exhibited superior killing compared to those generated using suboptimal purification strategies (Figure 7d). This enhanced transfection efficiency and cytotoxicity underscore the critical importance of utilizing high‐purity circRNA in T cell engineering. The superior functional quality of WMC‐purified circRNA is essential for maintaining CAR‐T cell functionality and viability, ultimately contributing to the effectiveness of CAR‐T cell therapies. Our findings emphasize that circRNA purity is a key factor in optimizing T cell engineering processes for biomedical applications.

## 3 Discussion

This study addresses the critical challenge of immunogenic impurities in synthetic circRNA, which can severely limit their therapeutic potential. Through comprehensive analysis, we identified key contaminants—such as dsRNA, RNA precursors, nicked RNA, and RNA phosphates—that significantly impact the immunogenicity and functional performance of circRNA. These impurities activate innate immune responses via RNA sensors, including PKR, OAS, TLR3, TLR7, TLR8, MDA5, and RIG‐I, triggering downstream signaling pathways such as NF‐κB, TNF, and TLR networks. Such immune activation not only reduces circRNA‐driven protein expression but also increases the risk of cellular dysfunction, highlighting the urgent need for effective purification methods to ensure the biosafety and functionality of circRNA therapeutics. Our findings demonstrate that even trace levels of these immunogenic impurities are sufficient to upregulate RNA sensors and inflammatory cytokines. As a result, achieving ultra‐pure circRNA is essential to minimize innate immune activation and ensure cell viability.

To address these issues, the study developed a novel purification strategy involving a multi‐step process combining enzymatic treatments and cellulose‐based filtration. This method effectively removes the identified immunogenic impurities, thereby reducing the activation of immune responses and significantly improving the manufacturing yields and purity of circRNA. The use of cellulose chromatography was particularly effective in selectively binding dsRNA, further reducing immunogenicity. This novel purification approach resulted in highly pure circRNA with significantly reduced immunogenicity. The improved production efficiency achieved by this purification approach is approximately tenfold higher than traditional size‐based methods, highlighting its potential for large‐scale manufacturing.

The findings of this study have significant implications for the production and application of circRNA in mRNA‐based cell programming. Our results emphasize the crucial importance of achieving high‐purity circRNA to maximize its functionality while minimizing cellular immunogenic responses. This is particularly beneficial for highly sensitive cells and challenging applications. Moreover, the integration of advanced purification techniques such as WMC chromatography with traditional methods could enhance the scalability and cost‐effectiveness of circRNA production. Continued investigation into the sources and impacts of RNA impurities will be essential to refine purification processes and improve the overall quality and safety of synthetic circRNA.

Future efforts should prioritize refining and simplifying the multi‐step purification process to facilitate broader industrial application. Developing a user‐friendly, single‐step purification method would be ideal for large‐scale manufacturing, significantly enhancing its industrial applicability. To achieve this, minimizing undesired byproducts in crude products is essential. Optimizing T7 polymerase to reduce dsRNA byproducts at the transcription source can alleviate downstream purification burdens, effectively addressing primary immunogenicity issues and improving both the safety and efficacy of the final product. Additionally, enhancing the efficiency of circRNA circularization will reduce linear RNA byproducts, further streamlining the purification process and improving overall manufacturing yields and purity. These strategic refinements are crucial for the scalable, cost‐effective production of high‐quality circRNA, which is vital for their successful biomedical and industrial applications.

In conclusion, this study provides a comprehensive approach to mitigating the immunogenicity of synthetic circRNA, paving the way for their safe and effective use in mRNA‐based cell programming. The novel purification strategy developed here represents a significant advancement in circRNA research, offering a robust solution to one of the key challenges in the field. The stepwise purification strategy developed in this study effectively addresses the challenge of immunogenic impurities, paving the way for safer and more effective circRNA‐based therapeutics, especially for complex therapeutic applications. By minimizing the activation of innate immune responses, this approach has the potential to enhance the biomedical translation of circRNA technology.

## 4 Experimental Section

### Cell Lines

Human cervical carcinoma cell lines (HeLa), A549, and Human monocytic cell line THP‐1 were obtained from the Cell Resource Center, Peking Union Medical College (which is the headquarter of National Science & Technology Infrastructure‐National BioMedical Cell‐Line Resource, NSTI‐BMCR). Peripheral Blood Mononuclear Cells (PBMCs) were purchased from Oricells Biotech (Shanghai, China). Human fibroblast cells and Raji cells stably transduced with luciferase (CTCC‐0326‐Luc2) were purchased from MEISEN CELL (Zhejiang, China).

HeLa and A549 cells were cultured at 37 °C and 5% CO_2_ in Dulbecco's Modified Eagle's Medium (4500 mg L^−1^ glucose) supplemented with 10% (v/v) heat‐inactivated fetal bovine serum (hi‐FCS, GIBCO) and 1% (v/v) penicillin/streptomycin (GIBCO). THP‐1 was maintained in RPMI 1640 GlutaMAX medium supplemented with 10% (v/v) hi‐FCS (FCS) and a 1% (v/v) mixture of penicillin and streptomycin in a humidified incubator containing 5% CO_2_ at 37 °C.

Primary human T cells were isolated from PBMCs using the RosetteSep Human T cell Enrichment kit (Stem Cell Technologies) according to the manufacturer's protocol. Cryopreserved T cells were thawed and activated on D0 with ImmunoCult Human CD3/CD28/CD2 T Cell Activator (Stem Cell Technologies) in ImmunoCult‐XF T Cell Expansion Medium (Stem Cell Technologies). Mycoplasma testing was conducted regularly.

### circRNA in Vitro Synthesis

circRNA precursors were synthesized by T7 High Yield RNA Transcription Kit (Vazyme, China) according to the manufacturer's protocols. After transcription, reactions were treated with DNase I (Vazyme, China) for 15 min. After DNase treatment, circRNA was incubated from reactions with 8 m LiCl. GTP was added to a final concentration of 2 mM along with a buffer including magnesium (50 mM Tris‐HCl, 10 mM MgCl_2_, 1 mM DTT, pH = 7.5; New England Biolabs) into circRNA solution. Reactions were then heated to 55 °C for 8 min. After circularization, 20 U RNase R and 10 × RNase R buffer (Epicentre) was added according to the manufacturer's protocols and were heated to 37 °C for 20 min. After RNase R digestion, circRNA was collected using LiCl precipitation. Then added 1 U µg^−1^ Quick CIP (New England Biolabs) and 10 × rCurSmart into circRNA and heated 37 °C for 20 min.

### FPLC Based Purification of circRNA

Two types of cellulose were used for circRNA purification: microcrystalline cellulose (MCC) and Natural Wood‐Derived Macroporous Cellulose (WMC).

For the MCC‐FPLC method, 450 mg of MCC was added to a 1 mL column, which was then connected to the FPLC system and washed with chromatography buffer (10 mM HEPES, 0.1 mm EDTA, 125 mm NaCl, and 25% or 30% (v/v) isopropanol) at a flow rate of 1 mL min^−1^ for 20 column volumes. Subsequently, 100 µg of circRNA was loaded into the column. After purification, the isopropanol‐containing eluent was precipitated with LiCl for recovery.

For WMC‐FPLC, the cellulose was synthesized as described in.^[^ ^34^ ^]^ 250 mg of WMC was added to a 1 mL column, and the WMC‐FPLC purification followed the same process using a different chromatography buffer (10 mm HEPES, 0.1 mm EDTA, 125 mm NaCl, and 16% (v/v) ethanol, pH 7.2).

### Spin Column Based Purification of circRNA

5.3 mg of WMC was filled into a spin column and washed using 500 µL elution buffer (10 mm HEPES, 0.1 mm EDTA, 125 mm NaCl, and 16% (v/v) ethanol, pH = 7.2) through vigorous shaking for 5 min. After 7000 rpm centrifugation for 10 s, the filtrate was discarded. Repeat the washing process several times, 10 µg of circRNA sample with 200 µL elution buffer was loaded into the column, and the mixture was shaken vigorously for 6 min. The unbound single‐stranded mRNA in the effluent was collected and separated with dsRNA by 14000 × g centrifugation for 1 min. For recovery, circRNA was precipitated with LiCl.

### SEC Purification of circRNA

circRNA was run through a 7.8 × 300 mm size exclusion column (Sepax Technologies; part number: 215950–7830) on AKTA FPLC (Cytiva). RNA was run in RNase‐free Potassium Phosphate buffer (10 mm potassium phosphate, 1 mm EDTA, pH = 6) at a flow rate of 0.4 mL min^−1^. For recovery, circRNA was precipitated with LiCl.

### Capillary Electrophoresis

RNA was tested using the 5200 Fragment Analyzer system (Agilent) with the Fragment Analyzer RNA Kits (DNF‐471‐0500) according to the manufacturer's instructions.

### HPLC

circRNAs were analyzed by an ion‐pair reversed‐phase (IP‐RP) HPLC with a 1260 system from Agilent Technologies. The analysis was conducted on a 250 × 4 mm reverse‐phase LC column with a particle size of 5 µm and a pore size of 4000 Å (Agilent Technologies: PLRP‐S 4000Å). A linear gradient was employed with buffer A (100 mm hexyl ammonium acetate [HAA] in 10% acetonitrile) and buffer B (100 mm HAA in 50% acetonitrile) at a flow rate of 0.4 mL min^−1^. The column oven temperature was maintained at 60 °C, and RNA was detected by UV absorbance at 260 nm.

### Lateral Flow Strip Assay

The test strip preparation followed the approach outlined in a previous report (31). 100 ng of circRNA was mixed with a 20% BSA (10 mm Tris‐HCl, pH = 8.0). The final total volume was 50 µL. The mixture was then added to the sample area of the test strip. After 15 min, the test strip was imaged on the ChemiDoc MP Imaging. The exposure time was set to 0.4 s.

### Cell Transfection

For HeLa cells, 400 ng of RNA was transfected into 4 × 10^4^ cells/200 µL per well of a 48‐well plate using Lipofectamine 3000 (Invitrogen) according to the manufacturer's instructions. To obtain PMA‐differentiated THP‐1 cells, THP‐1 cells were seeded at a density of 2.5 × 10^5^ cells/500 µL per well in a 12‐well culture plate and stimulated with 100 ng mL^−1^ of PMA for 48 h. A549 cells were seeded at a density of 2 × 10^5^ cells/500 µL per well in a 12‐well culture plate 24 h prior to transfection. For fibroblasts, 10^4^ cells/200 µL per well 24 before transfections. A total of 1 µg or 200 ng of RNA, or 40 ng of HMW or intron fraction was transfected into A549, fibroblasts, and THP‐1 cells. All the cells were transfected using Lipofectamine 3000 (Invitrogen) according to the manufacturer's instructions.

For PBMCs, 1 µg of RNA was added to 2 × 10^5^ cells re‐suspended in 10 µL of T buffer R from the Neon Transfection System 10 µL Kit (Invitrogen). Electroporation was performed at 1600 V, 10 ms, 3 pulses settings using a Neon electroporation device (Invitrogen). Upon electroporation, the cells were seeded on the recovery plate with T cell media and 100 IU mL^−1^ of IL‐2.

### circRNA Reprogramming

Fibroblasts were plated on dishes coated with matrigel. (Corning)following the manufacturer's instructions, at densities of 6000 cells per well in a 6‐well dish format. The cells were cultured in reproTESR (Stemcell)″ supplemented with 200 ng mL^−1^ B18R (novo protein). The medium was replaced daily, both before and after circRNA transfections. B18R supplementation was discontinued the day after the final transfection. Different rounds of: 24‐h transfections with circRNA were performed as previously described^[^ ^35^ ^]^ using LipoMessengerMAX (Invitrogen)according to the manufacturer's instructions. Each transfection delivered 800 ng of' circRNA per well, containing Oct4, Sox2, Klf4, c‐Myc, Lin28A, and Nanog at·a molecular ratio of 3:1:1:1:1:1.

### Cellular Fluorescence and Flow Cytometry

24 h after RNA transfection, cells were imaged on a fluorescence microscope (OLYMPUS CKX53). For eGFP detection, cells were washed three times with PBS and resuspended in 100 µL of PBS before analysis on a flow cytometer (ACEA NovoCyte, Agilent). For surface staining, T cells were washed twice in FACS buffer (2% FBS in PBS) before staining with fluorescent‐labeled antibodies. The cells were resuspended in 100 µL of antibody solutions and incubated at 4 °C for 30 min. After incubation, the cells were washed twice and resuspended in 100 µL of PBS before analysis on a flow cytometer (ACEA NovoCyte, Agilent). Data were analyzed using FlowJo software version 10.8. Transfection efficiency was quantified as the percentage of GFP‐positive cells relative to the total cell population. The expression level of GFP mRNA was represented by the MFI of fluorescein isothiocyanate (FITC) in positive cells. For CAR expression level, cells were prepared in staining buffer (PBS supplemented with 2% FBS) and subjected to fluorochrome‐conjugated antibody (APC‐Labeled Monoclonal Anti‐FMC63 Antibody, Acro, FM3‐AY54A1) staining for surface markers, following standard protocols.

### Cytotoxicity Studies

The cytotoxicity studies were conducted according to.^[^ ^39^ ^]^ Raji–luc target cells were pelleted with CAR‐T cell products in 96‐well plates at effector: target (CAR^+^ T‐cell: Raji–luc) ratios of 10:1, 5:1, 2.5:1, 1:1, and 0:1 for 20 h. 1% triton X‐100 was used as a positive control. 1 × 10^5^ CAR^+^ T cells and 1 × 10^4^ target cells were used as the highest effector‐to‐target ratio in a total volume of 100 µL RPMI supplemented with 10% FBS. After 20 h, CAR‐T cells and Raji–luc co‐cultures were lysed and the luminescence signal of the solution was quantified using a SpectraMAX M5 fluorescence microplate reader (Molecular Device). Percent cytotoxicity was calculated as 1 − (sample − ctrl_triton X‐100_)/(ctrl_0:1_ − ctrl_triton X‐100_)%.

### RNA Isolation and RT‐qPCR

Total RNAs were extracted with FastPure Cell/Tissue Total RNA Isolation Kit V2 (Vazyme, China) according to the manufacturer's protocol. 0.1 mg of total RNAs were served as the template for reverse transcription by using PrimeScript II 1st Strand cDNA Synthesis Kit (TAKARA) according to the manufacturer's protocols. After reverse transcription, cDNAs were transcribed with Taq Pro Universal SYBR qPCR Master Mix (Vazyme, China) according to the manufacturer's protocol. Primer pairs were selected using the Primer Blast designing tool (https://www.ncbi.nlm.nih.gov/tools/primer‐blast/). Relative expression of mRNA abundance was determined from three independent experiments and was normalized by housekeeping gene B2 m or GAPDH.

### RNA Sequencing

The total RNA of each sample was isolated and purified using Trizol reagent (Invitrogen, Carlsbad, CA, USA) following the manufacturer's procedure. The RNA amount and purity of each sample were quantified using NanoDrop 2000 (NanoDrop, Wilmington, DE, USA). The RNA integrity was assessed by Agilent 2100 with an RIN number >7.0. Then, the mRNA of each sample was enriched using oligo(dT) magnetic beads. RNA‐seq libraries with an average insert size of 350 bp were constructed using AHTS Universal V8 RNA‐seq Library Prep Kit for Illumina (Vazyme, China) following the manufacturer's instructions, and then sequenced on SURFSeq 5000 platform (GeneMind Biosciences LTD., China) with PE150 model. SOAPnuke^[^ ^40^ ^]^ was adopted to remove the reads with an adaptor, ploy‐N, and low‐quality bases (‐n 0.01 ‐l 15 ‐q 0.5 –ada_trim –minReadLen 75 –polyX 50). The Cleaned reads were then aligned to the human reference genome (GRCh38) with Histat2.^[^ ^41^ ^]^ The mapped reads were delivered to StringTie to perform expression quantification.^[^ ^42^ ^]^ Differentially expressed genes/transcripts (DEGs/DETs) were identified with p.value < 0.05 and |log2(fold change) | ≥1 by the R package DESeq2.^[^ ^43^ ^]^ The KEGG and GO enrichment of DEGs was conducted with the R package clusterProfiler^[^ ^44^ ^]^ with *p*.value < 0.05 as the threshold for significantly enriched DEGs.

### Statistics Analysis

Data were presented as mean ± standard deviation (SD). The specific sample size (n) for each experiment is reported in the figure legends. Statistical significance was assessed using one‐way ANOVA, with a significance level set at *p* < 0.05. Any assumptions required for the chosen statistical analysis were checked before testing. All analyses were conducted using GraphPad Prism 9.0.

## Conflict of Interest

The authors declare no conflict of interest.

## Supporting information



Supporting Information

## Data Availability

The data that support the findings of this study are available from the corresponding author upon reasonable request.
