# Supplementary material for: Mitigating Cellular Dysfunction Through Contaminant Reduction in Synthetic circRNA for High‐Efficiency mRNA‐Based Cell Reprogramming
Source: Adv Sci (Weinh). 2025 Mar 5;12(16):2416629. doi: 10.1002/advs.202416629 (PMC12021033; doi:10.1002/advs.202416629)
Supplement: Supplementary file 1 — Supporting Information [file ADVS-12-2416629-s001.docx]

Mitigating cellular dysfunction through contaminant reduction in synthetic circRNA for high-efficiency mRNA-based cell reprogramming

**Authors**

Ziwei Zhang^1,7^, Weiyu Li^1,2,7^, Dengwang Luo^1,3^, Xiangyu Ren^1, 6^, Xiushuang Yuan^1^, Li Yu^1,4^, Daming Wang^5^, Yuhong Cao^1,6^*

Supplemental Figures and Tables


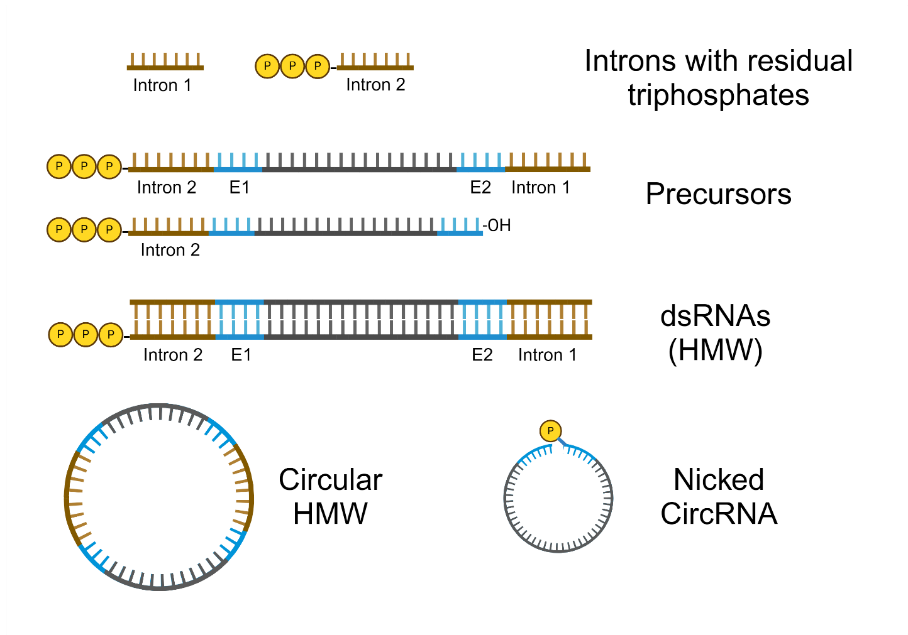


**Fig. S1. The byproducts in synthetic circRNA may elicit innate immune response, including dsRNA, 5’ triphosphates introns and precursors, and hydrolyzed circRNA (nicked RNA). (Created with BioRender.com)**


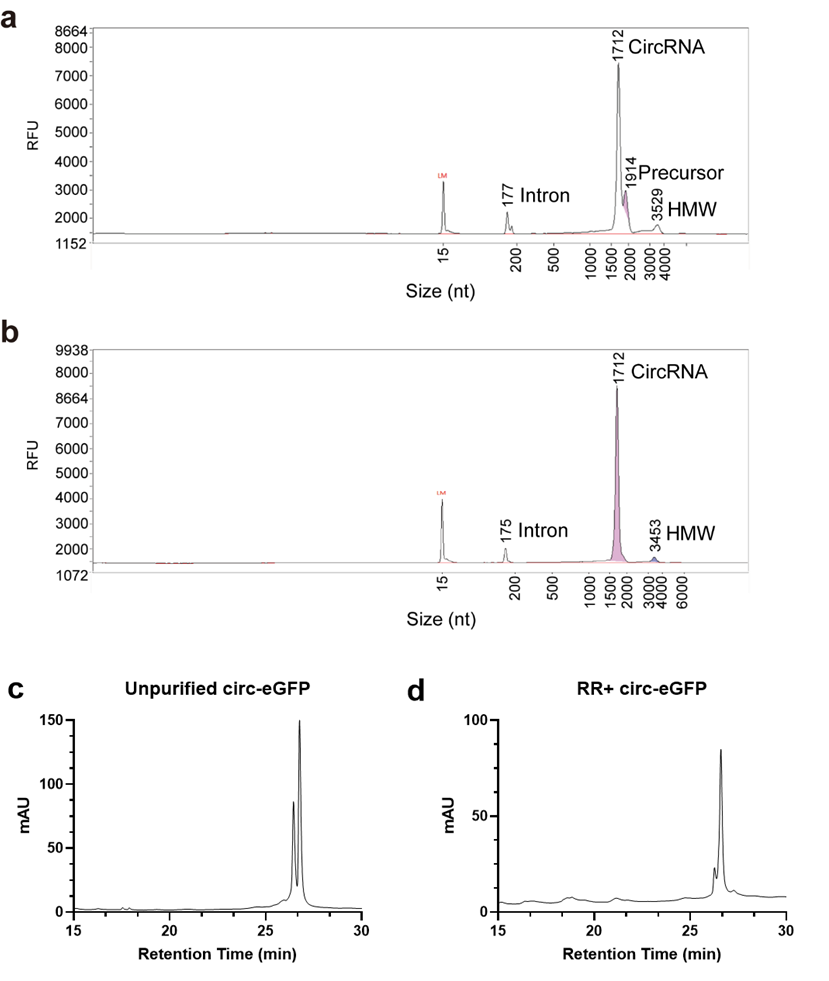


Fig. S2. Capillary electrophoresis analysis of circ-eGFP.

**a & c.** IVT-synthesized circ-eGFP (unpurified).

**b & d.** IVT-synthesized circ-eGFP after RNase R treatment (RR+).


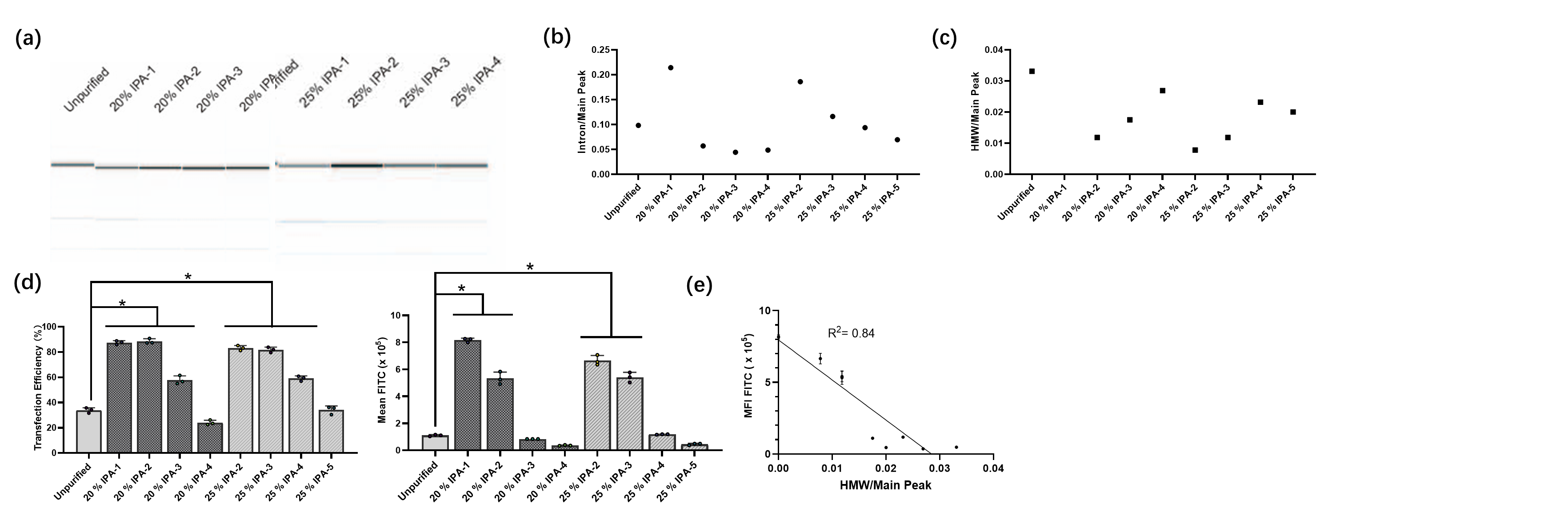


Fig. S3.

a. MCC chromatogram of RR+ circ-eGFP under 25 % (blue) IPA HEPES equilibrium conditions.

Capillary electrophoresis of circRNA fractions.

b-c. Distribution of intron and HMW byproducts across different fractions as detailed in Figure 3b, respectively.

d. Translation efficiency and mean fluorescence intensity (MFI) 24 hours post-transfection of HeLa cells with different fractions as outlined in a. Data presented as means ± SDs of three biological replicates, *p < 0.05, according to Student's t-test.

e. Linear correlation between MFI and HMW ratio.


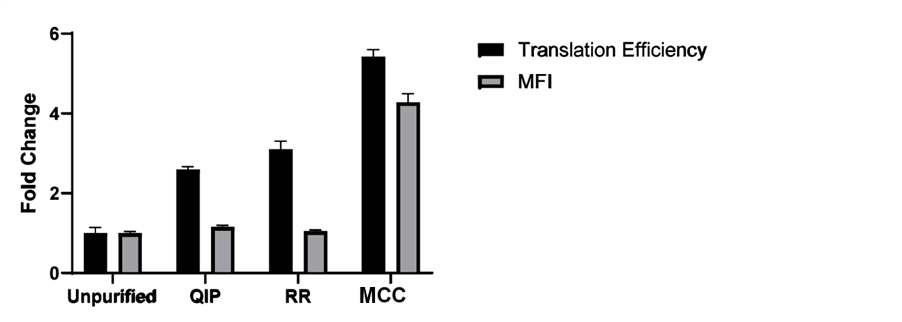


Fig. S4. Relative translation efficiency and Mean Fluorescence Intensity of circ-eGFP with different treatments (QIP: quick phosphatase, RR, Rnase R, MCC chromatography) to the unpurified circ-eGFP.


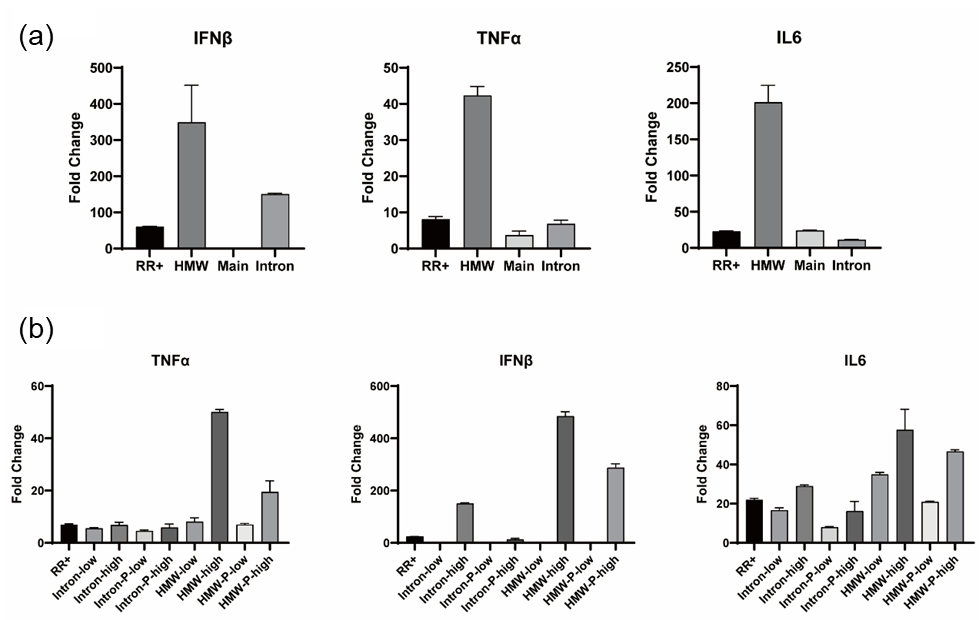


Fig. S5. Immunogenicity assessment of circ-eGFP fractions with enzymatic treatments.

1. Induction of IL6, TNFα and IFNβ transcripts 6 hours after transfection of 40 ng of HMW or introns, 200 ng of RR+circ-eGFP, main fraction into 0.2 million A549 cells, respectively. B2M mRNA was used as a housekeeping control. Data presented as means ± SDs, n = 3.
2. Induction of IL6, TNFα and IFNβ transcripts 6 hours after transfection of A549 cells with the 40 ng or 200 ng of intron and HMW before and after phosphatase treatment (intron-P, HMW-P), or 200 ng of main fraction after phosphatase treatment (main-P). B2M mRNA was used as a housekeeping control. Data presented as means ± SDs, n = 3.


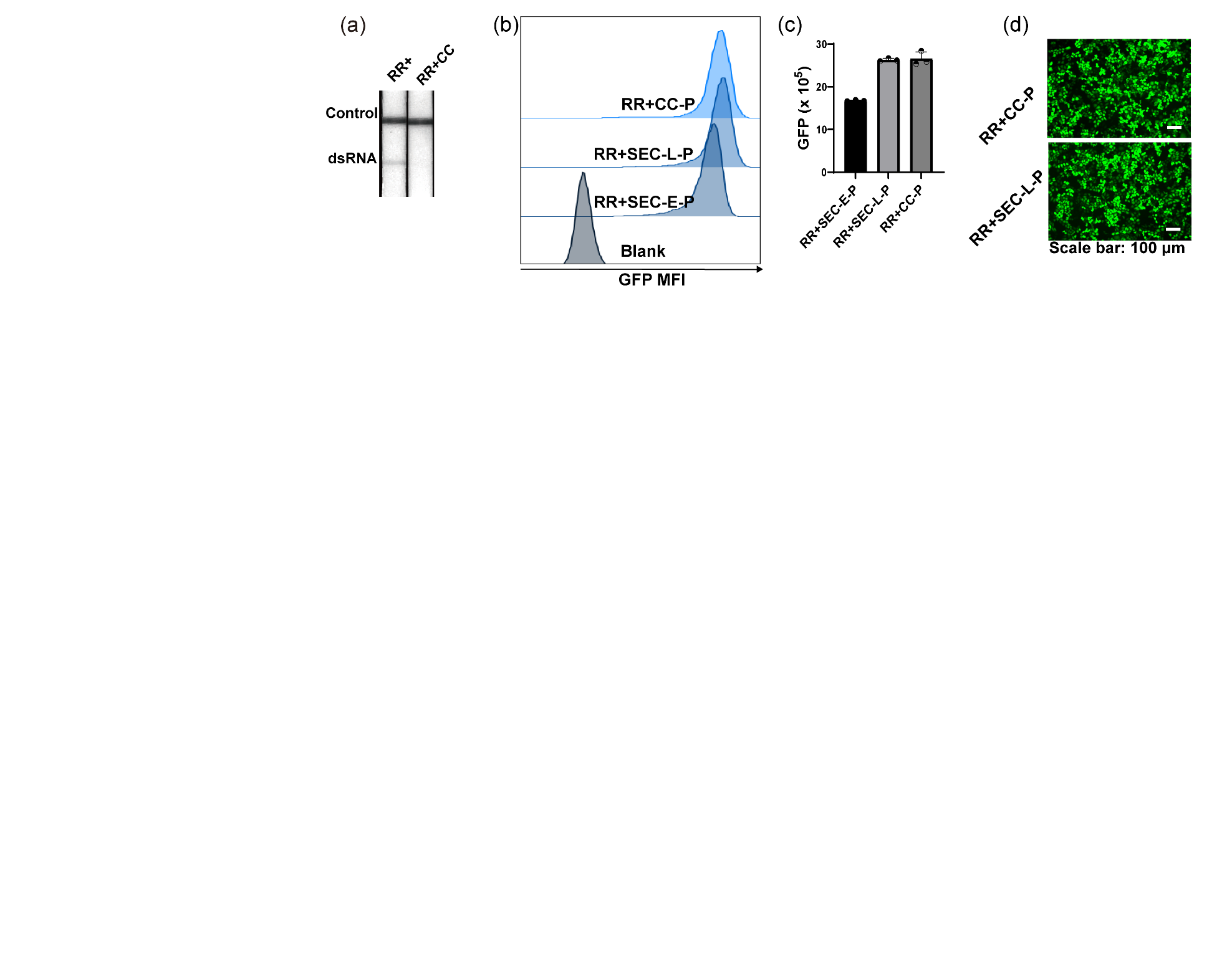


**Fig. S6. Combination of RR digestion, cellulose chromatography (CC) and phosphatase achieve high translability.**

1. LSFA assessment of RR+ circRNA before and after CC.

**b-d.** Representative intensity and MFI of eGFP, and cellular fluorescence image (scale bar: 100 μm) captured 24 hours post-transfection of Hela cells with RR **and** RR**+**CC-P circ-eGFP.


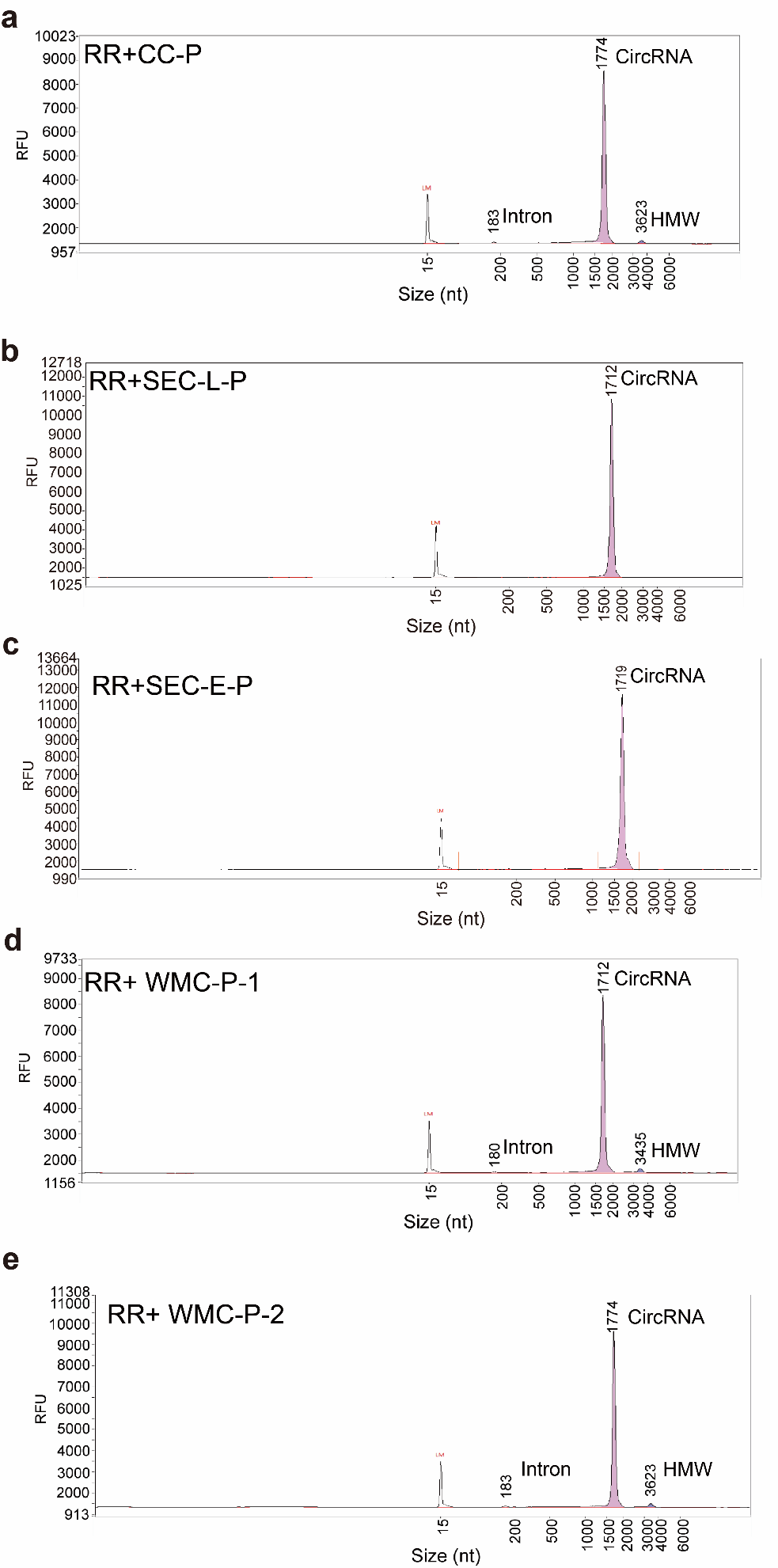


Fig. S7. Capillary electrophoresis of circ-eGFP with different purification methods.


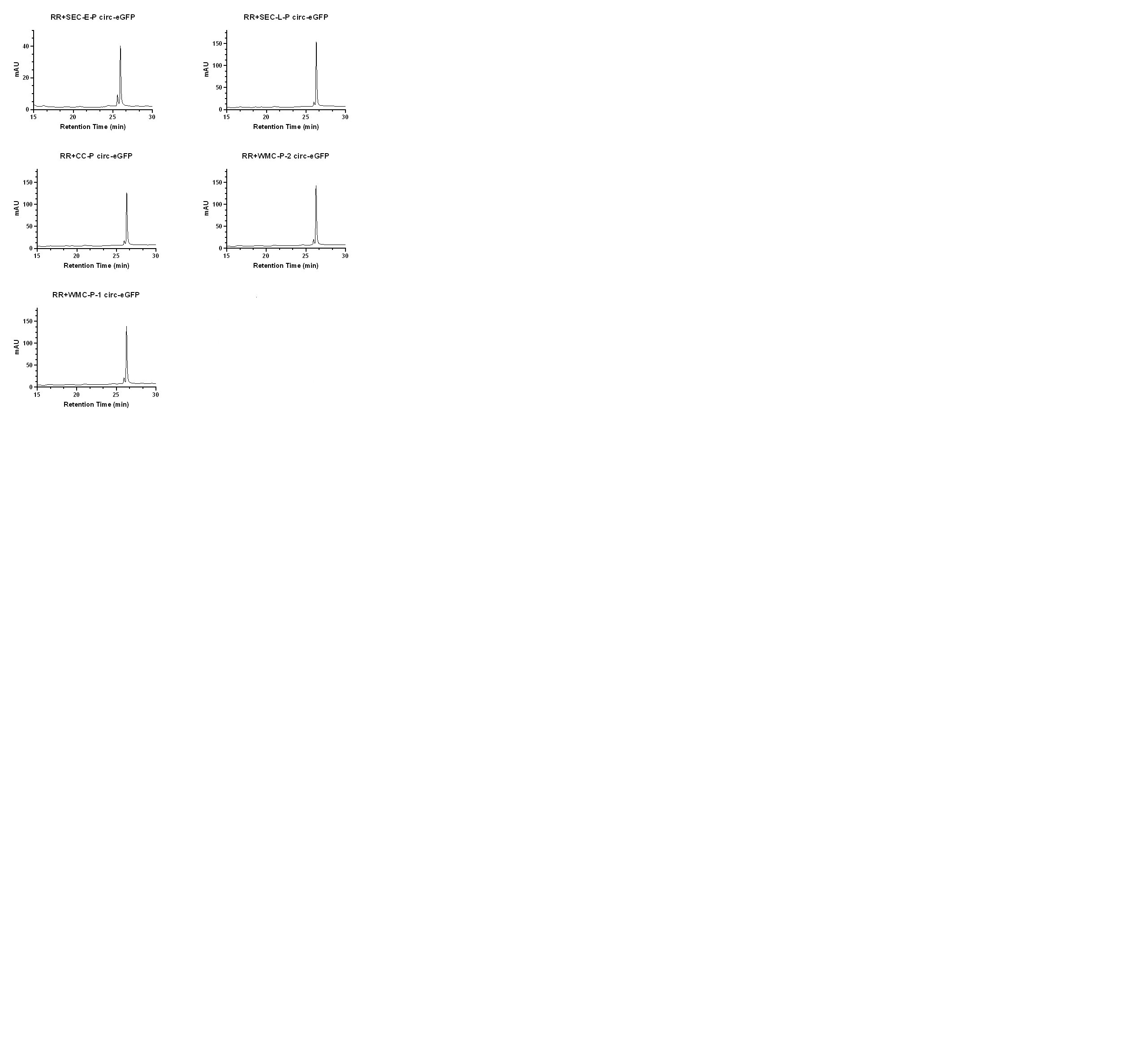


Fig. S8. IP-RP-HPLC of circ-eGFP with different purification methods.


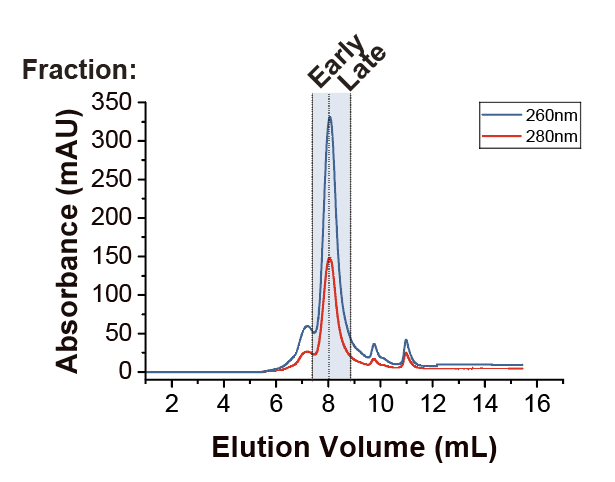


**Fig. S9. SEC early and late peaks circRNA.**


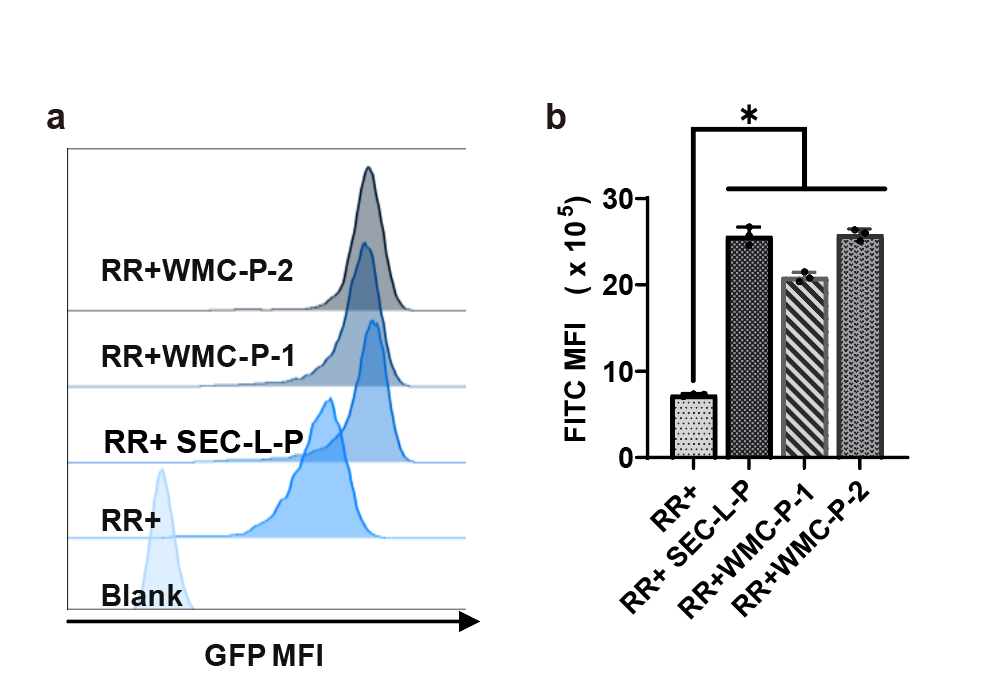


Fig. S10. WMC chromatography to purify circRNA.

1. Protein expression level 24 hours after transfection of HeLa cells with RR+, RR+SEC-L-P, RR+WMC-P-1 and RR+WMC-P-2 circ-eGFP.
2. MFI 24 hours after transfection of HeLa cells with RR+, RR+SEC-L-P, RR+WMC-P-1 and RR+WMC-P-2 circ-eGFP. Data presented as means ± SDs, n = 3; One-way ANOVA, *p<0.05.


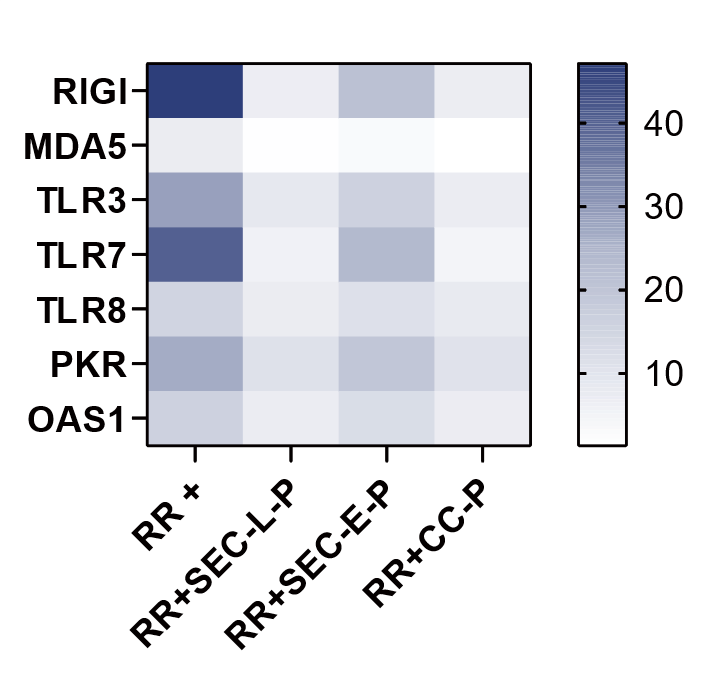


**Fig. S11 Expression levels of immune response genes (RIG-I, TLR3, TLR7, PKR, OAS1, and MDA5) in PMA-differentiated THP-1 cells 6 hours post-transfection with circRNA purified by different methods Data normalized to B2M and presented as mean ± SDs (n = 3).**


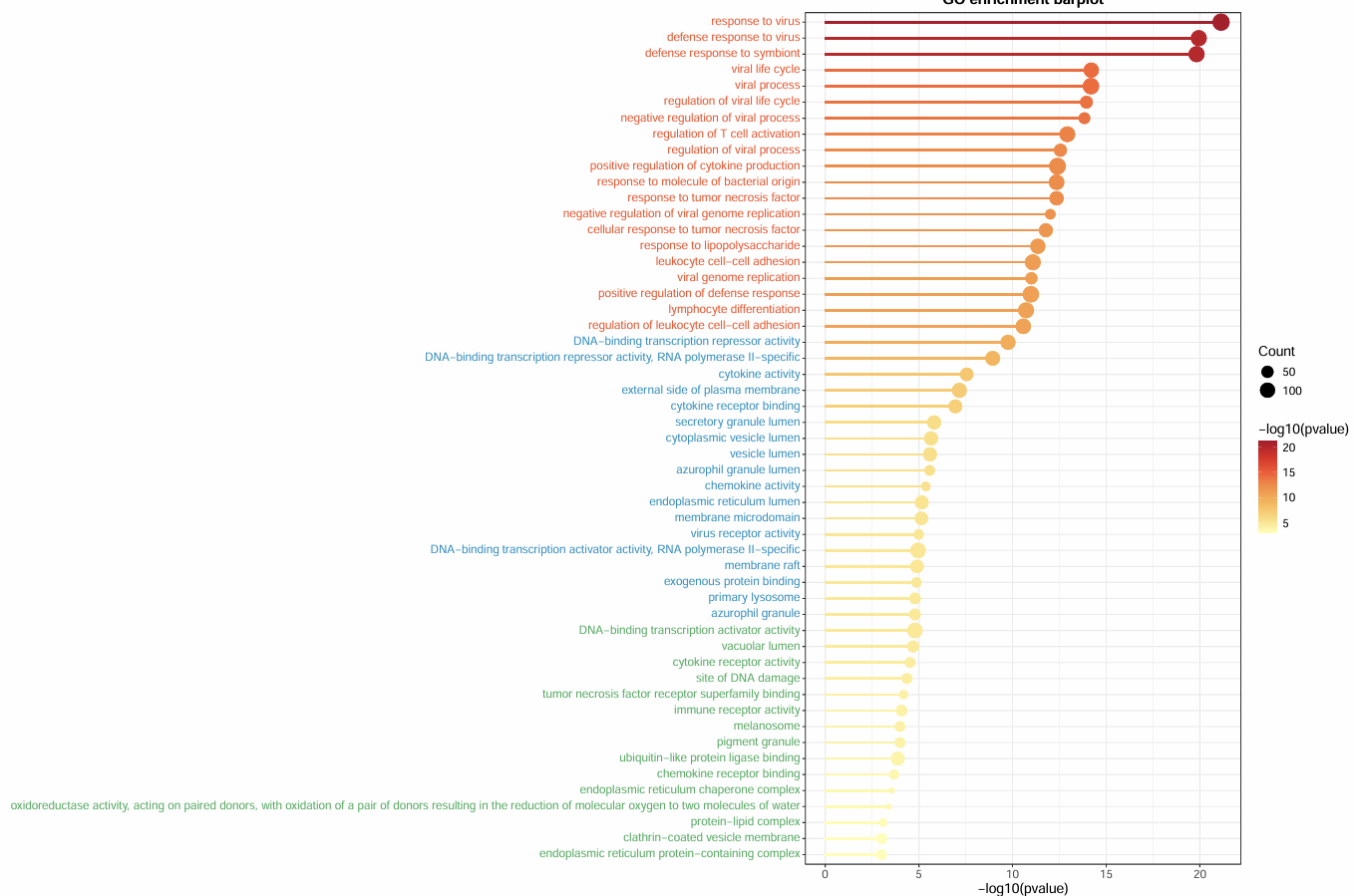


Fig. S12. GO enrichment analysis between PMA-differentiated THP-1 cells transfected HMW-CC and HMW.


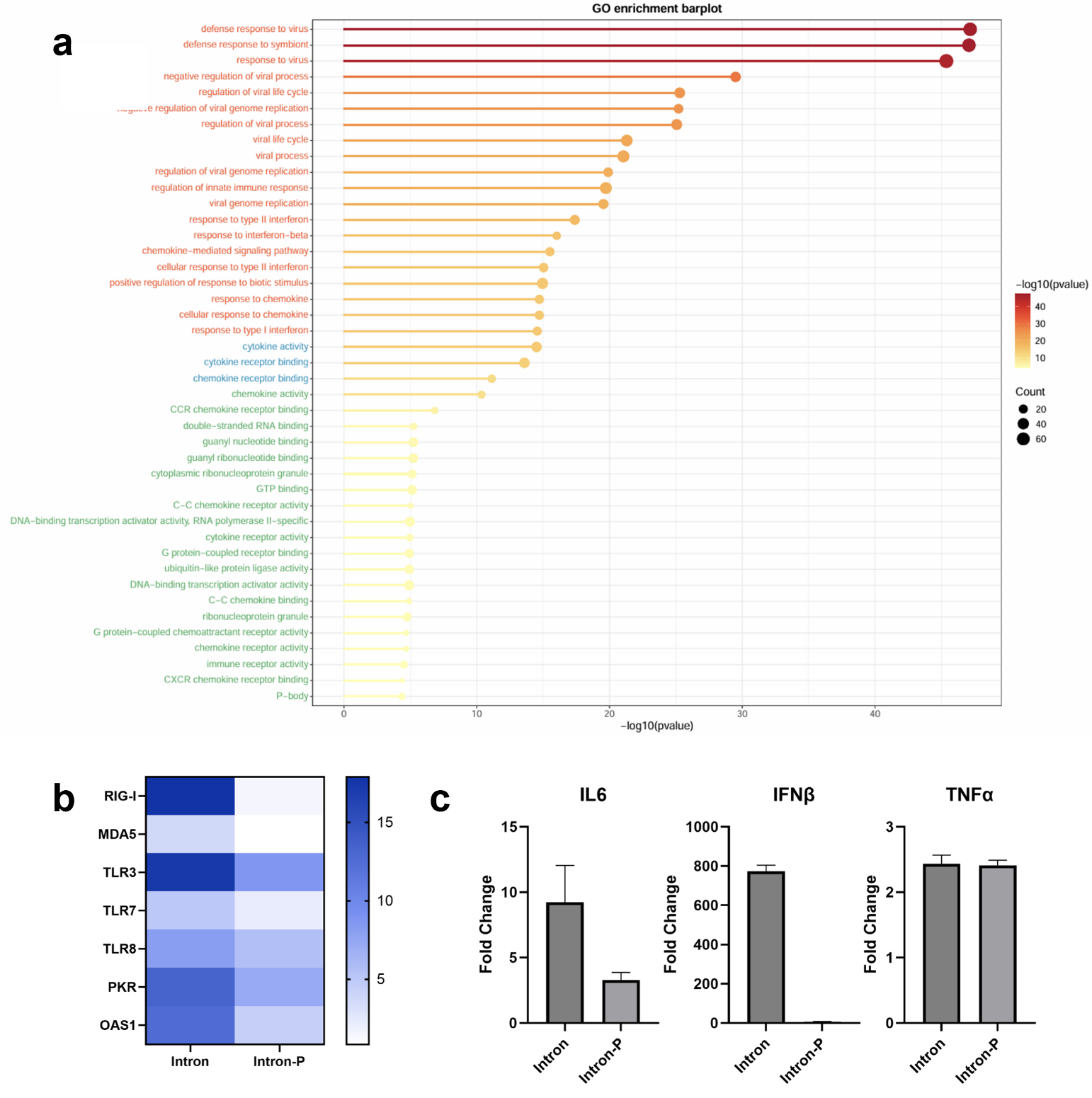


Fig. S13. Immunogenicity assessment of intron before and after phosphatase treatment.

**a** GO enrichment analysis between PMA-differentiated THP-1 cells transfected with intron after phosphatase treatment (intron-P) and intron.

**b** Induction of RIG-I, MAD5 and TLR3, TLR7 transcripts 6 hours after transfection of intron or intron-P into PMA-differentiated THP-1 cells. B2M mRNA was used as a housekeeping control. Data presented as a means of three biological replicates.

**c** Induction of IL6, TNFα and IFNβ transcripts 6 hours after transfection of intron or intron-P into PMA-differentiated THP-1 cells. B2M mRNA was used as a housekeeping control. Data presented as means ± SDs of three biological replicates.


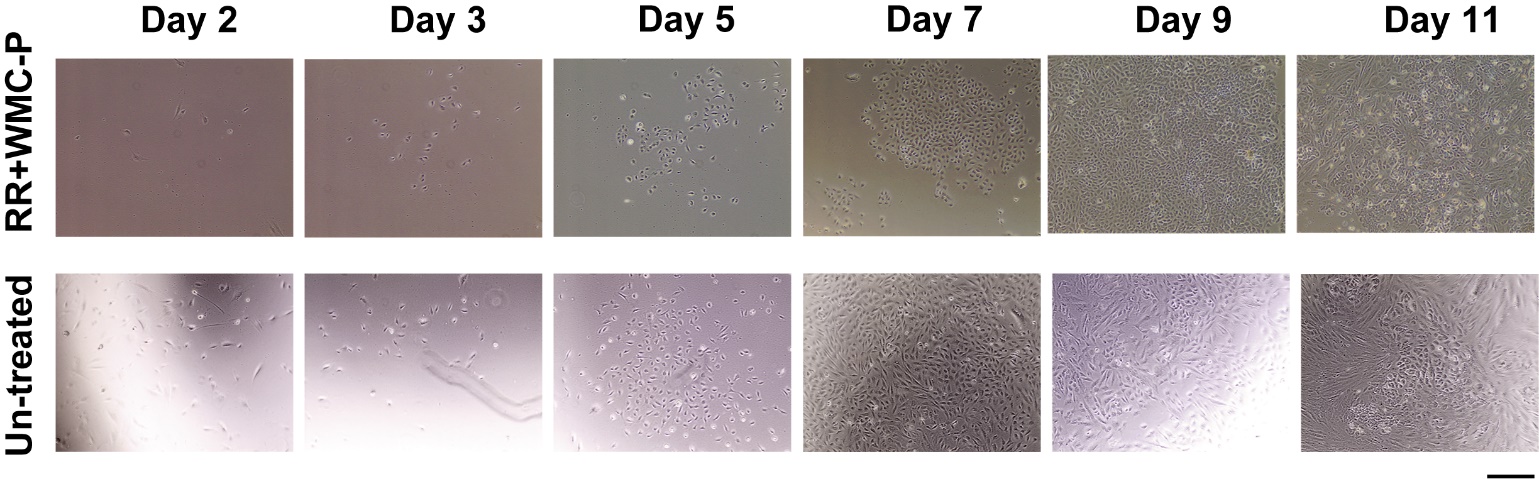


**Fig. S14.** Morphological changes observed during the RR+WMC-P circRNA-based reprogramming regimen with three transfections, initiated with a plating density of 1,000 cells (scale bar: 300 μm).

Table S1 Intron and HMW proportions to main peak of different fractions in Figure 3A.

| **Sample** | **Intron/Main Peak** | **HMW/Main Peak** |
| --- | --- | --- |
| Unpurified | 0.098 | 0.033 |
| 20 % IPA-1 | 0.214 | Not Detected |
| 20 % IPA-2 | 0.057 | 0.012 |
| 20 % IPA-3 | 0.044 | 0.018 |
| 20 % IPA-4 | 0.049 | 0.027 |
| 25 % IPA-2 | 0.186 | 0.008 |
| 25 % IPA-3 | 0.116 | 0.012 |
| 25 % IPA-4 | 0.094 | 0.023 |
| 25 % IPA-5 | 0.070 | 0.020 |

Table S2 Reagents and resources

| **Chemicals, Peptides, and Recombinant Proteins** | **SOURCE** | **IDENTIFIER** |
| --- | --- | --- |
| PMA | Sigma | Catalog # P1585 |
| Lipo3000 | Invitrogen™ | Catalog # L3000015 |
| Human recombinant IL-2 | STEMCELL^TM^ | Catalog # 78145.1 |
| ImmunoCult™ Human CD3/CD28 T Cell Activator | STEMCELL^TM^ | Catalog # 17951 |
| ImmunoCult™-XF T Cell Expansion Medium | STEMCELL^TM^ | Catalog # 10981 |
| RNase R | Epicentre | Catalog # RNR07250 |
| DNase I | Vazyme | Catalog # EN402-01 |
| Quick CIP | New England Biolabs | Catalog # M0525V |
| HEPES | Solarbio | Catalog # 7365-45-9 |
| NaCl | Solarbio | Catalog # 7647-14-5 |
| EDTA | Thermo Fisher | Catalog # J15694 |
| IPA | Innochem | Catalog # I1700 |
| Potassium Phosphate Buffer | Bioleaper | Catalog # BR4000306 |
| **Critical Commercial Assays** |  |  |
| Neon™ Transfection System 10 μL Kit | Invitrogen™ | Catalog # MPK1096 |
| T7 High Yield RNA Transcription Kit | Vazyme | Catalog # TR101-01 |
| FastPure Cell/Tissue Total RNA Isolation Kit V2 | Vazyme | Catalog # RC112-01 |
| PrimeScript™ II 1st Strand cDNA Synthesis Kit | TAKARA | Catalog # 6210A |
| Taq Pro Universal SYBR qPCR Master Mix | Vazyme | Catalog # Q712-02 |
| 5 moU eGFP mRNA | Vazyme |  |

**Table S3 Primers**

| **Primers** |  |
| --- | --- |
| B2M-F | AGGACTGGTCTTTCTATCTC |
| B2M-R | TTCATCCAATCCAAATGCGG |
| IL6-F | GACTGCAGGAACTCCTTAAAGC |
| IL6-R | GAGTAGTGAGGAACAAGCCAGAG |
| IFNβ-F | TTGAATGGGAGGCTTGAATACT |
| IFNβ-R | TAGCCAGGAGGTTCTCAACAAT |
| TNFα-F | AAGACCACCACTTCGAAACCT |
| TNFα-R | AGGCCTAAGGTCCACTTGTGT |
| TLR7-F | TGCTGTGTGGTTTGTCTGGTG |
| TLR7-R | CTCCTGGCCCCACACAAGT |
| TLR8-F | TCCGCACTTGAAACTAAGACC |
| TLR8-R | CTGGCACAAATGACATCTAC |
| OAS1-F | TGGATTCTGCTGGTGAGACC |
| OAS1-R | ATGGCCTTTGGCAAGAGGTAAG |
| RIG-I-F | AAATCAGAACACAGGCAGAGGAA |
| RIG-I-R | GTCCCATGTCTGAAGGCGTAA |
| MDA5-F | GCATATGCGCTTTCCCAGTG |
| MDA5-R | CTCTCATCAGCTCTGGCTCG |
| PKR-F | TCCATGGGGAATTACATAGGC |
| PKR-R | AGCGGCCAATTGTTTTGCTT |
| TLR3-F | CCTTTTGCCCTTTGGGATGC |
| TLR3-R | TGAAGTTGGCGGCTGGTAAT |
| Oct4-F | GGAGGAAGCTGACAACAATGAAA |
| Oct4-R | GGCCTGCACGAGGGTTT |

Table S4 RNA sequences

| **Gene** | **RNA sequences** |
| --- | --- |
| eGFP | ATGGTGAGCAAGGGCGAGGAGCTGTTCACCGGGGTGGTGCCCATCCTGGTCGAGCTGGACGGCGACGTAAACGGCCACAAGTTCAGCGTGTCCGGCGAGGGCGAGGGCGATGCCACCTACGGCAAGCTGACCCTGAAGTTCATCTGCACCACCGGCAAGCTGCCCGTGCCCTGGCCCACCCTCGTGACCACCCTGACCTACGGCGTGCAGTGCTTCAGCCGCTACCCCGACCACATGAAGCAGCACGACTTCTTCAAGTCCGCCATGCCCGAAGGCTACGTCCAGGAGCGCACCATCTTCTTCAAGGACGACGGCAACTACAAGACCCGCGCCGAGGTGAAGTTCGAGGGCGACACCCTGGTGAACCGCATCGAGCTGAAGGGCATCGACTTCAAGGAGGACGGCAACATCCTGGGGCACAAGCTGGAGTACAACTACAACAGCCACAACGTCTATATCATGGCCGACAAGCAGAAGAACGGCATCAAGGTGAACTTCAAGATCCGCCACAACATCGAGGACGGCAGCGTGCAGCTCGCCGACCACTACCAGCAGAACACCCCCATCGGCGACGGCCCCGTGCTGCTGCCCGACAACCACTACCTGAGCACCCAGTCCGCCCTGAGCAAAGACCCCAACGAGAAGCGCGATCACATGGTCCTGCTGGAGTTCGTGACCGCCGCCGGGATCACTCTCGGCATGGACGAGCTGTACAAGTAA |
| circRNA scaffold | GGGAGACCCTCGACCGTCGATTGTCCACTGGTCAACAATAGATGACTTACAACTAATCGGAAGGTGCAGAGACTCGACGGGAGCTACCCTAACGTCAAGACGAGGGTAAAGAGAGAGTCCAATTCTCAAAGCCAATAGGCAGTAGCGAAAGCTGCAAGAGAATGAAAATCCGTTGACCTTAAACGGTCGTGTGGGTTCAAGTCCCTCCACCCCCACGCCGGAAACGCAATAGCCGAAAAACAAAAAACAAAAAAAACAAAAAAAAAACCAAAAAAACAAAACACAAAAAAACAAAAAACAAAACGGCTATTATGCGTTACCGGCGAGACGCTACGGACTTAAATAATTGAGCCTTAAAGAAGAAATTCTTTAAGTGGATGCTCTCAAACTCAGGGAAACCTAAATCTAGTTATAGACAAGGCAATCCTGAGCCAAGCCGAAGTAGTAATTAGTAAGACCAGTGGACAATCGACGGATAACAGCATATCTA |
| **Cvb3** | **TTAAAACAGCCTGTGGGTTGATCCCACCCACAGGCCCATTGGGCGCTAGCACTCTGGTATCACGGTACCTTTGTGCGCCTGTTTTATACCCCCTCCCCCAACTGTAACTTAGAAGTAACACACACCGATCAACAGTCAGCGTGGCACACCAGCCACGTTTTGATCAAGCACTTCTGTTACCCCGGACTGAGTATCAATAGACTGCTCACGCGGTTGAAGGAGAAAGCGTTCGTTATCCGGCCAACTACTTCGAAAAACCTAGTAACACCGTGGAAGTTGCAGAGTGTTTCGCTCAGCACTACCCCAGTGTAGATCAGGTCGATGAGTCACCGCATTCCCCACGGGCGACCGTGGCGGTGGCTGCGTTGGCGGCCTGCCCATGGGGAAACCCATGGGACGCTCTAATACAGACATGGTGCGAAGAGTCTATTGAGCTAGTTGGTAGTCCTCCGGCCCCTGAATGCGGCTAATCCTAACTGCGGAGCACACACCCTCAAGCCAGAGGGCAGTGTGTCGTAACGGGCAACTCTGCAGCGGAACCGACTACTTTGGGTGTCCGTGTTTCATTTTATTCCTATACTGGCTGCTTATGGTGACAATTGAGAGATCGTTACCATATAGCTATTGGATTGGCCATCCGGTGACTAATAGAGCTATTATATATCCCTTTGTTGGGTTTATACCACTTAGCTTGAAAGAGGTTAAAACATTACAATTCATTGTTAAGTTGAATACAGCAAA** |
